# *Magel2* truncation alters select behavioral and physiological outcomes in a rat model of Schaaf-Yang syndrome

**DOI:** 10.1242/dmm.049829

**Published:** 2023-02-03

**Authors:** Derek L. Reznik, Mingxiao V. Yang, Pedro Albelda de la Haza, Antrix Jain, Melanie Spanjaard, Susanne Theiss, Christian P. Schaaf, Anna Malovannaya, Theresa V. Strong, Surabi Veeraragavan, Rodney C. Samaco

**Affiliations:** ^1^ Baylor College of Medicine, Department of Molecular and Human Genetics, Houston, TX 77030, USA; ^2^ Texas Children's Hospital, Jan and Dan Duncan Neurological Research Institute, Houston, TX 77030, USA; ^3^ Baylor College of Medicine, Mass Spectrometry Proteomics Core, Houston, TX 77030, USA; ^4^ Heidelberg University, Institute of Human Genetics, Im Neuenheimer Feld 366, 69120 Heidelberg, Germany; ^5^ Baylor College of Medicine, Verna and Marrs McLean Departments of Biochemistry and Molecular Biology, and Molecular and Cellular Biology, Houston, TX 77030, USA; ^6^ Baylor College of Medicine, Dan L. Duncan Comprehensive Cancer Center, Houston, TX 77030, USA; ^7^ Foundation for Prader-Willi Research, Walnut, CA 91789, USA; ^8^ Department of Genetics, University of Alabama at Birmingham, Birmingham, AL 35294, USA

**Keywords:** Schaaf-Yang syndrome, Prader-Willi syndrome, *Magel2*, Imprinting, Rat model

## Abstract

Previous studies in mice have utilized *Magel2* gene deletion models to examine the consequences of its absence. We report the generation, molecular validation and phenotypic characterization of a novel rat model with a truncating *Magel2* mutation modeling variants associated with Schaaf-Yang syndrome-causing mutations. Within the hypothalamus, a brain region in which human MAGEL2 is paternally expressed, we demonstrated, at the level of transcript and peptide detection, that rat *Magel2* exhibits a paternal, parent-of-origin effect. In evaluations of behavioral features across several domains, juvenile *Magel2* mutant rats displayed alterations in anxiety-like behavior and sociability measures. Moreover, the analysis of peripheral organ systems detected alterations in body composition, cardiac structure and function, and breathing irregularities in *Magel2* mutant rats. Several of these findings are concordant with reported mouse phenotypes, indicating the conservation of MAGEL2 function across rodent species. Our comprehensive analysis revealing impairments across multiple domains demonstrates the tractability of this model system for the study of truncating *MAGEL2* mutations.

## Teaser

**Summary:** The molecular, neurobehavioral and organ system characterization of a rat model of Schaaf-Yang syndrome provides insight into the consequences of a truncating mutation in the imprinted gene *Magel2*.

## INTRODUCTION

The neurodevelopmental disorders (NDDs) Schaaf-Yang syndrome [SYS; Online Mendelian Inheritance in Man (OMIM) #615547], Chitayat-Hall syndrome and Opitz trigonocephaly C syndrome (OMIM #211750) share overlapping clinical features that have been attributed to commonly shared loss-of-function truncating mutations in the imprinted gene *MAGEL2*. MAGEL2 is enriched in the brain, and heterozygous mutations in the active paternal copy of *MAGEL2* leave individuals vulnerable to neurological dysfunction as the maternal allele is epigenetically silenced (Boccaccio et al., 1999; Schaaf et al., 2013). Previous studies into the consequences of MAGEL2 deficiency have focused on its complete loss of function, as *MAGEL2* is also a gene frequently deleted in patients with Prader-Willi syndrome (PWS; OMIM #176270).

Mice carrying deletions of the paternal *Magel2* have been the primary mammalian system used to examine the biological systems and molecular pathways affected by the absence of MAGEL2 (Bervini and Herzog, 2013). Although current murine deletion models may be effective tools to study the consequences of the complete absence of MAGEL2, they may also limit our knowledge on the evaluation of consequences that are directly linked to *MAGEL2* truncating mutations. Moreover, owing to an increased prevalence of intellectual disability, autism spectrum disorder (ASD) and disrupted social behaviors in people with SYS and MAGEL2-related disorders compared to PWS (McCarthy et al., 2018; Patak et al., 2019; Marbach et al., 2020), evaluating the consequences of truncated MAGEL2 in species that enable the study of more complex behavioral patterns could provide deeper insight for the study of these specific phenotypes.

One approach to complement studies of mouse models for understanding rare NDDs is the laboratory rat, a genetically tractable system with greater genetic conservation to the human genome (Gibbs et al., 2004). The behavioral repertoire of the rat includes sophisticated social and cognitive behaviors (Pellis and Pellis, 1998; Rudy, 2009). The innate difference in sociability of rats compared to that of mice (Pellis and Pasztor, 1999) presents a unique opportunity for research into the behavioral features of NDDs. Beyond direct consequences of the central nervous system (CNS), people with an NDD also frequently suffer from dysfunction of peripheral organ systems. In the added context of therapeutic development, the large overall size of the rat offers unique advantages in preclinical proof-of-concept validation studies (Wöhr and Scattoni, 2013); combined with techniques that are readily accessible to manipulate the rat genome, the laboratory rat enables a level of study that can enhance ongoing efforts for the study of and development of treatments for rare genetic NDDs. To this end, the purpose of our study was to identify the neurobehavioral, organ system and molecular consequences of a truncating *Magel2* mutation in the rat. By using an evolutionarily more complex mammalian system than the mouse, we sought out to identify changes in surrogate phenotypic domains that may be pathogenically relevant to mechanistic pathways underlying the consequences of *MAGEL2* truncating mutations.

## RESULTS

### Transcription activator-like effector nuclease (TALEN) targeting of rat *Magel2* results in a predicted truncating mutation that does not affect levels of mRNA abundance

**Fig. 1.**
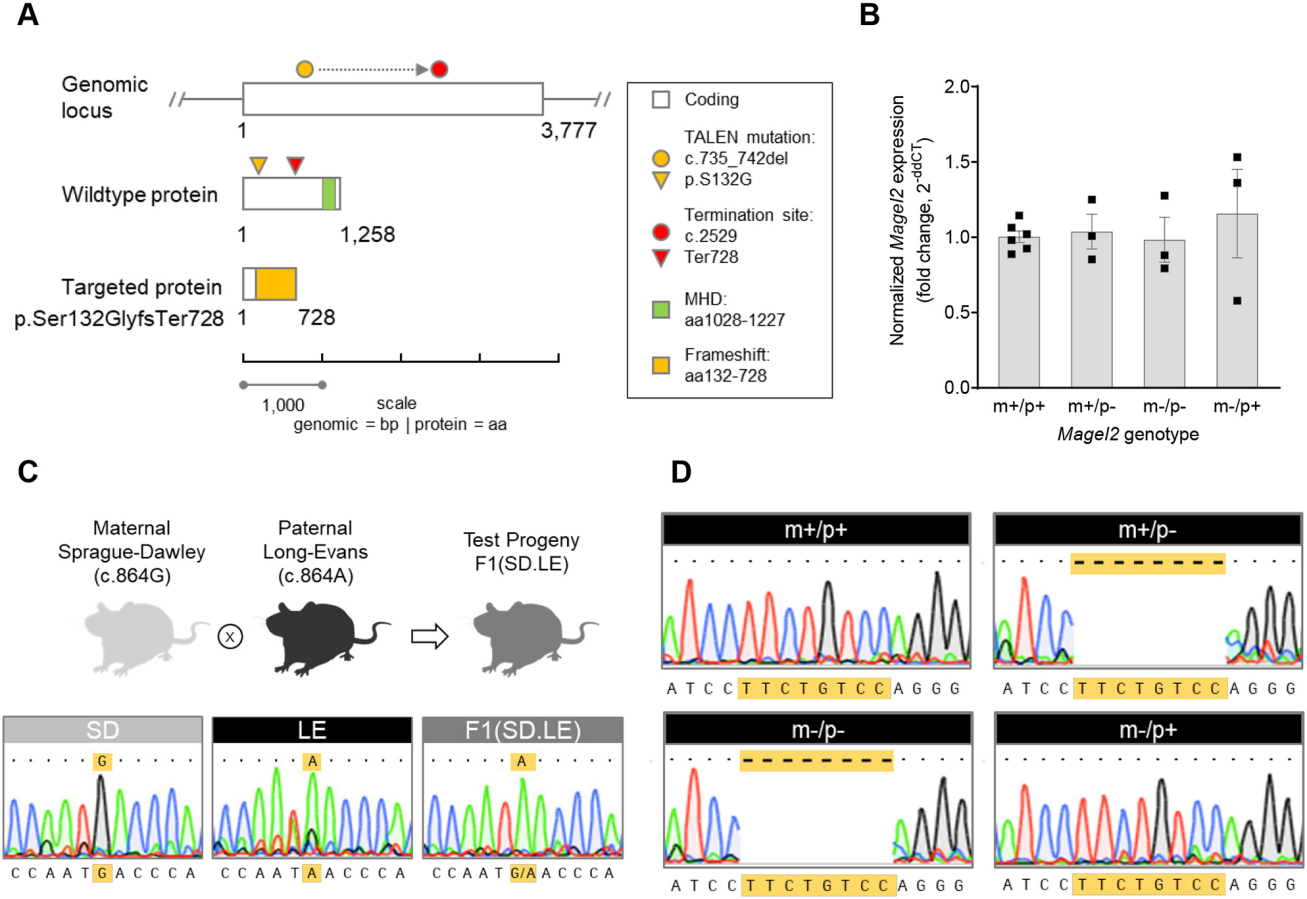
**Strategy and validation of a novel *Magel2* rat model**. (A) Diagram showing the overall strategy to create a *Magel2* rat model. Rat *Magel2*: single-exon gene located on chromosome 1 (genomic locus, white bar; 3777 bp), protein product [wild-type protein, white bar; 1258 amino acids (aa)] with a MAGE homology domain (MHD; green bar). Transcription activator-like effector nuclease (TALEN) modification of the wild-type locus created an 8 bp deletion (c.735_742; yellow circle), predicted to shift the open-reading frame and result in loss of full-length MAGEL2 protein (targeted protein, p.Ser132Glyfs728). Additional predicted features of the 8 bp-induced mutation include translational of a novel aa sequence (targeted protein, yellow bar) and loss of the conserved MHD. (B) Relative *Magel2* mRNA abundance measured by qRT-PCR showed no difference in *Magel2* expression levels in mutant genotype groups compared to wild-type littermate rats. Allelic inheritance and corresponding genotype of each *Magel2* group is shown as either maternal (m) or paternal (p), and wild-type (+) or mutant (−). Hypothalamic tissue samples from rats heterozygous for the paternally or maternally inherited mutant *Magel2* allele (m+/p− or m−/p+, respectively), rats homozygous for both mutant alleles (m−/p−) and wild-type rats (m+/p+) were used. Data are depicted as mean±s.e.m.; individual data points are denoted by black squares. *n*=3-6/rats per genotype group. (C) Endpoint RT-PCR and sequencing of hypothalamic cDNA from parental rat strains [maternal Sprague-Dawley (SD); paternal Long-Evans (LE)] and F1 intercross hybrid progeny (F1.SD.LE) confirms a parent-of-origin effect on rat *Magel2* expression. The wild-type *Magel2* coding sequence contains a single-nucleotide polymorphism (SNP) at position c.864 that differs between SD (c.864G) and LE (c.864A) rats. Representative sequence traces of the SNP from parental and F1 hybrid genotypes is shown (yellow highlight); F1(SD.LE) hybrid rats show expression of paternal LE-derived SNP, c.864A. Rat images created with BioRender.com. (D) Endpoint RT-PCR and sequencing of hypothalamic cDNA from rats derived from the *Magel2* mutant model validate a parent-of-origin effect on rat *Magel2* expression in the context of the TALEN-modified *Magel2* rat allele (wild-type littermates, m+/p+; heterozygous *Magel2* rats, m+/p− and m−/p+; homozygous rats, m−/p−). Representative sequence traces show each *Magel2* genotype sequence aligned to the rat reference sequence. Matching sequences (dots) in each window are denoted. m+/p+ and m−/p+ show complete alignment. The absence of sequence (dash, highlighted in yellow) corresponding to the TALEN-generated 8 bp deletion was found only in *Magel2* genotype groups with paternal inheritance of the mutation (m+/p− and m−/p−).

An 8 bp deletion was generated in the single-exon coding sequence of the rat *Magel2*, c.735_742del, and was confirmed with sequencing. The deletion results in a frameshift event causing a change from serine to glycine at amino acid position 132; in turn, a mutant peptide sequence extends through to a premature termination site at amino acid 728, resulting in a predicted truncated MAGEL2 (Fig. 1A). Given that *Magel2* is enriched in the hypothalamus, we focused on its expression within this brain region. To enable a complete readout of total mRNA abundance, we used primers that spanned a region after the TALEN-targeted mutation site in *Magel2* to amplify a product commonly shared between the mutant and wild-type alleles independent of potential MAGEL2 parent-of-origin effects. Relative mRNA abundance was comparable in the hypothalami of rats representing all four possible *Magel2* genotypes, demonstrating that nonsense-mediated decay was not occurring. Heterozygous rats with either the paternally or maternally inherited *Magel2* TALEN-generated mutation (*Magel2^m+/p−^*, indicated as m+/p−; and *Magel2^m−/p+^*, indicated as m−/p+), as well as homozygous rats with biparental inheritance of the *Magel2* TALEN-generated mutation (*Magel2^m−/p−^*), showed normal *Magel2* mRNA abundance in comparison to that in wild-type rats (Fig. 1B), suggesting that inheritance of the mutation does not affect expression levels.

### Rat *Magel2* transcript exhibits parent-of-origin-specific expression

Several rat loci are known to be imprinted (Berg et al., 2020; Overall et al., 1997); however, *Magel2* has not been evaluated in rats even though it is maternally imprinted in both mice and humans (Boccaccio et al., 1999). Given the absence of altered mRNA abundance in the *Magel2* TALEN-generated rat model (Fig. 1), we reasoned that an evaluation of parent-of-origin effects would yield important control evidence to test the validity of the rat model. Therefore, to determine whether *Magel2* is paternally expressed in the rat, we leveraged a naturally occurring single-nucleotide polymorphism (SNP) identified between two strains of rats at nucleotide (nt) position 864 (Rat Genome Database, Rnor 5.0; Smith et al., 2020) to enable analysis of parent-of-origin expression in progeny derived from Long-Evans (LE) male rats mated to Sprague-Dawley (SD) female rats. The 864th nt in SD rats is a guanine, whereas that in LE rats is an adenine. By amplifying a product spanning this region followed by Sanger sequencing, we found that hypothalamic *Magel2* shows a pattern of paternal inheritance and expression in F1 hybrid test progeny, indicated by an adenine at nt position 864 (Fig. 1C). Using the identical endpoint real-time reverse transcriptase PCR (RT-PCR) approach, we tested whether the *Magel2* mutant rats in either the heterozygous or homozygous mutation state showed a similar parent-of-origin, paternal-specific expression pattern. cDNA sequencing of mutant and wild-type hypothalami showed that the *Magel2* mutation was indeed preferentially expressed from the paternal allele, as evident in *Magel2^m+/p−^* (denoted as m+/p−) and *Magel2^m−/p−^* (denoted as the m−/p−) progeny with paternal inheritance of the *Magel2* mutation (Fig. 1D). In comparison, chromatograms from *Magel2^m+/p+^* progeny with inheritance of inheritance of wild-type parental *Magel2* alleles (denoted as m+/p+), and *Magel2^m−/p+^* with maternal inheritance of the *Magel2* mutation (denoted as m−/p+) (Fig. 1D), showed normal cDNA sequence traces. Taken together, these control studies confirm that *Magel2* is preferentially expressed from the paternally inherited allele across all genotypes. Moreover, expression from the maternally inherited allele in the rat hypothalamus was undetectable by Sanger sequencing, suggesting that the total mRNA abundance measured by quantitative RT-PCR (qRT-PCR) reflected expression levels specifically from the paternally expressed *Magel2.*

### Paternal heterozygous or homozygous loss of *Magel2* in juvenile rats results in altered perseverative-repetitive behavior, anxiety-like behavior and sociability

The primary goal of our phenotype profiling was to test the hypothesis that rats with the paternally inherited *Magel2* mutation represent a construct-valid model to study outcomes that emerge as a consequence of truncated MAGEL2. To this end, independent cohorts were generated to first evaluate test progeny derived from breeding schemes producing rats with the paternally inherited *Magel2* mutation (*Magel2^m+/p−^*, denoted as m+/p−), followed by examination of homozygous, biparental inheritance of the *Magel2* mutation (*Magel2^m−/p−^*, denoted as m−/p−). Wild-type littermates were also generated within each breeding scheme and evaluated as control comparisons. To test the neurobehavioral consequences of the truncating *Magel2* mutation in the rat, we deployed a well-defined battery of test procedures to assess for alterations across multiple domains, including anxiety-like behavior, sociability, locomotor function, sensorimotor gating, perseverative behavior, learning and memory, and pain nociception (Veeraragavan et al., 2016).

**Fig. 2.**
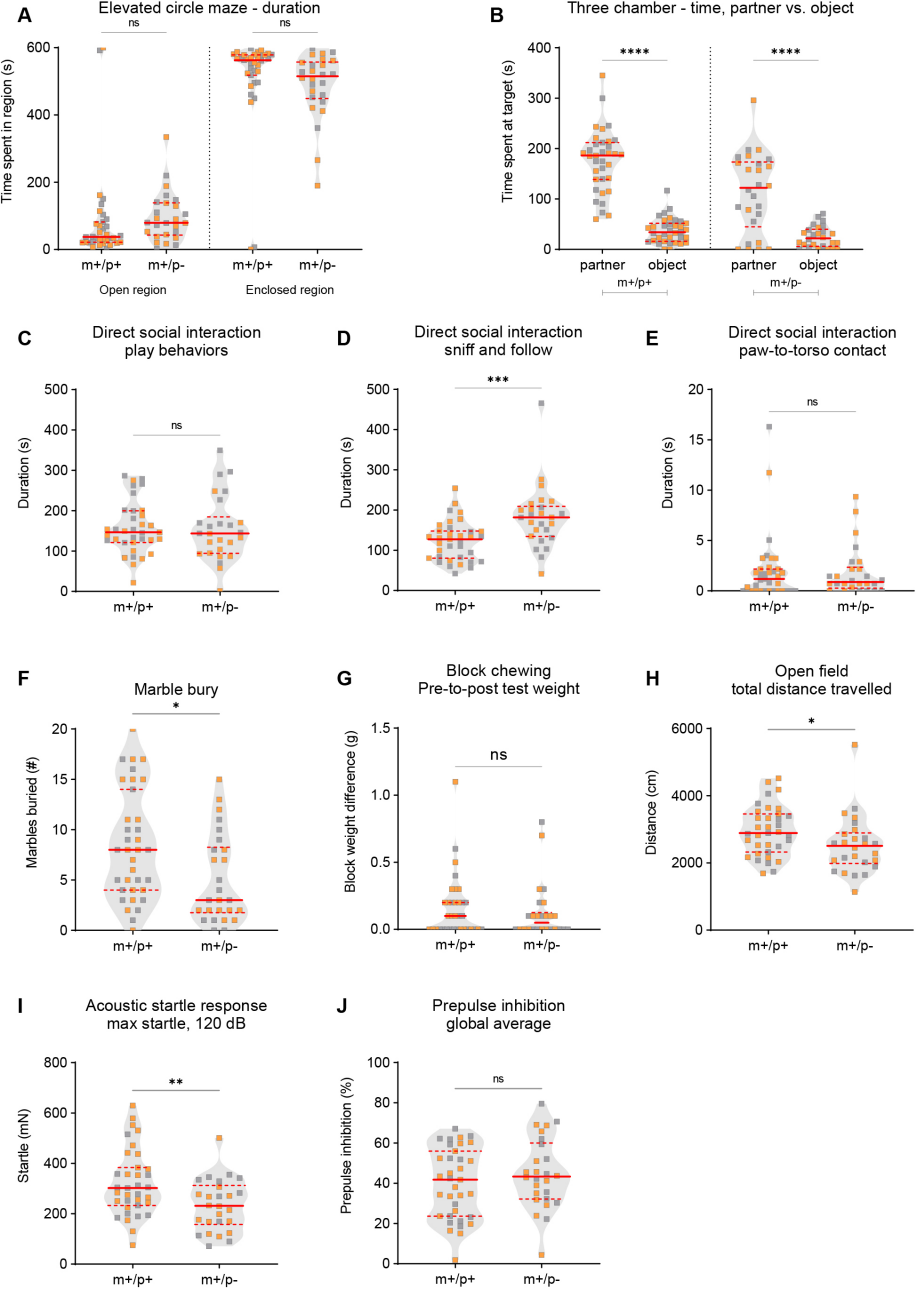
***Magel2^m+/p−^* rats display select alterations in dyadic social interaction, perseverative-like behavior, locomotor activity and startle response.** (A) *Magel2^m+/p−^* rats (m+/p−) compared to wild-type littermates (m+/p+) spent a comparable amount of time in the open and enclosed regions of the elevated circle maze. (B) *Magel2^m+/p−^* rats and wild-type littermates spent a statistically significant amount of time investigating conspecific partners in comparison to the novel object in the three-chamber test for social approach. (C-E) In a test of juvenile dyadic social interaction, *Magel2^m+/p−^* rats, in comparison to wild-type littermates, showed a comparable amount of time engaged in play-like behavior (C), a statistically significant increase in the duration of sniffing and following conspecific partners (D), and a comparable time spent in general paw-to-torso contact (E). (F,G) In tests of perseverative-like behavior, *Magel2^m+/p−^* rats buried fewer marbles (F); no differences were observed in the block-chew test (G). (H) *Magel2^m+/p−^* rats showed a significant decrease in total distance traveled. (I,J) Acoustic startle response at 120 dB in *Magel2^m+/p−^* rats was significantly reduced (I); prepulse inhibition of the startle response was normal (J). Violin plots were used to indicate data density and distribution, with gray (males) and orange (females) squares within each genotype/group category; solid red lines indicate the median and dashed red lines indicate quartiles. *P<0.05; **P<0.01; ***P<0.001; ****P<0.0001; ns, not significant; mutant rats relative to wild-type littermate controls; repeated measures (RM) ANOVA or multivariate two-way ANOVA using genotype and sex as main effects followed by post-hoc analyses were conducted when appropriate. A statistical summary of behavioral data is provided in Table S1.

*Magel2^m+/p−^* rats displayed significantly altered behavioral outcomes in select social and perseverative behaviors (Fig. 2). In the elevated circle maze test for anxiety-like behavior (Braun et al., 2011), *Magel2^m+/p−^* rats, in comparison to wild-type littermates, showed normal exploratory activity in the open and enclosed regions of the elevated circle maze (Fig. 2A). In the three-chamber test for social approach (Ku et al., 2016; Veeraragavan et al., 2016; Yang et al., 2011), the typical expected pattern of social approach behavior was observed – spending significantly more time investigating a novel conspecific partner compared to a novel object (Fig. 2B). To further investigate sociability, we evaluated dyadic direct social play behaviors. In contrast to the laboratory mouse that has been reported to display only rudimentary forms of play-like behavior, direct social interaction with dyad pairs of rats enables the study of developmentally restricted social behaviors uniquely observed in juvenile aged rats (Pellis and Pellis, 1998; Veeraragavan et al., 2016). We quantified play-like behavior, sniffing and following behavior, and general contact defined as test subject paw to conspecific subject torso (paw-to-torso contact) interaction in dyad interactions between either mutant or wild-type rats with wild-type non-littermate same-sex rat conspecific partners. Among these three behavioral categories, we found that *Magel2^m+/p−^* rats, in comparison to wild-type littermates, showed no difference in the duration of play-like behavior, a significant increase in the duration of sniffing and following conspecific wild-type non-littermate partner rats, and normal duration of general contact behaviors (Fig. 2C-E). Similarly, the number of observed events during juvenile dyadic interactions was comparable across genotypes for play-like and general contact behaviors, and significantly increased for sniffing and following behavior in *Magel2^m+/p−^* rats, in comparison to that in wild-type littermates (Fig. S1A-C). To evaluate perseverative, repetitive-like behavior, we examined the performance of *Magel2^m+/p−^* rats and their wild-type littermates in the marble-burying test and in an overnight assessment of the block-chew test (Hamilton et al., 2014; Veeraragavan et al., 2016). *Magel2^m+/p−^* rats displayed a significant reduction in the number of marbles buried (Fig. 2F), with no difference in the block-chew test (Fig. 2G). To measure spontaneous locomotor exploratory activity in a novel environment, *Magel2^m+/p−^* rats, in comparison to wild-type littermates, showed a modest but significant decrease in total distance traveled in the open-field assay (Fig. 2H). Other measures of generalized locomotor activity in the open-field test, including center distance traveled, vertical activity and the ratio of center-to-total distance traveled, were comparable across genotype groups (Fig. S1D-F). To evaluate sensorimotor gating, we tested prepulse inhibition (PPI) of the acoustic startle response in rats presented with a randomized series of low-decibel sound levels immediately preceding a high-decibel sound (120 dB). *Magel2^m+/p−^* rats, in comparison to wild-type littermates, displayed a significant reduction in baseline startle reactivity to the high-decibel sound (Fig. 2I); however, PPI of the startle response was normal (Fig. 2J).

**Fig. 3.**
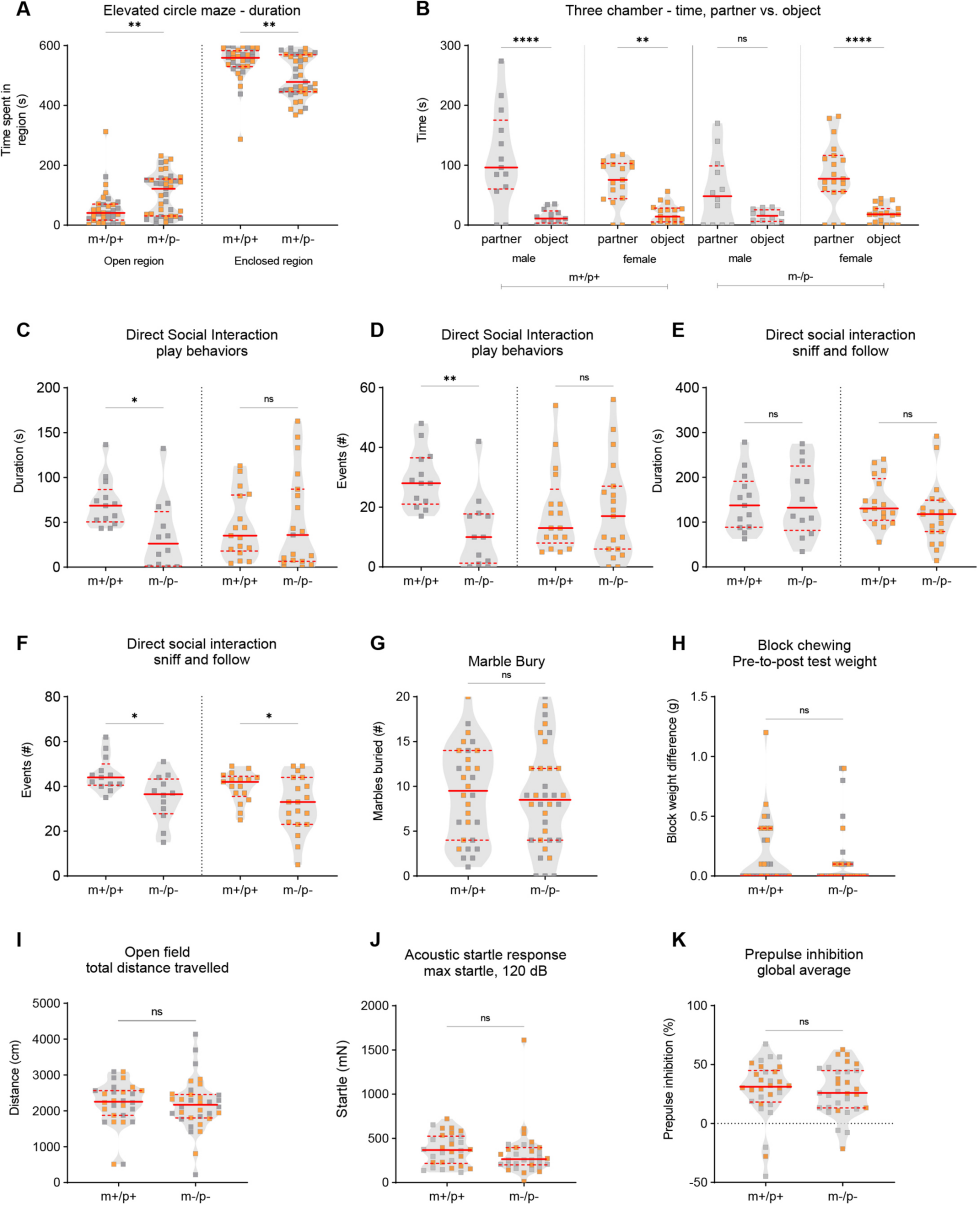
***Magel2^m−/p−^* rats display select alterations in anxiety-like behavior, social approach and dyadic social interaction.** (A) *Magel2^m−/p−^* rats (m−/p−), compared to wild-type littermates (m+/p+), spent more time in the open regions and less time in the closed regions in the elevated circle maze. (B) Wild-type littermates and *Magel2^m−/p−^* female rats spent significantly more time investigating a partner versus an object in the three-chamber test; in contrast, the times male *Magel2^m−/p−^* rats investigated the partner versus the object were not significantly different. (C-F) In dyadic social interaction tests, male, but not female, *Magel2^m−/p−^* rats showed a reduction in play-like behavior duration and number of events (C,D). In addition, although male and female *Magel2^m−/p−^* rats showed normal sniff and follow duration (E), both groups showed a significantly reduced number of sniff and follow events (F). (G,H) In tests for perseverative-like behavior, *Magel2^m−/p−^* rats showed no significant differences in marble burying (G) or block chewing (H). (I) Total distance traveled in the open field was comparable across genotypes. (J,K) Acoustic startle reactivity to a loud decibel noise (120 dB) (J) and prepulse inhibition of the startle response (K) were normal in *Magel2^m+/p−^* rats. Violin plots were used to indicate data density and distribution, with gray (males) and orange (females) squares within each genotype/group category; solid red lines indicate the median and dashed red lines indicate quartiles. *P<0.05; **P<0.01; ****P<0.0001; ns, not significant; mutant rats relative to wild-type littermate controls; RM ANOVA or multivariate two-way ANOVA using genotype and sex as main effects followed by post-hoc analyses were conducted when appropriate. A statistical summary of behavioral data is provided in Table S1.

Homozygous *Magel2^m−/p−^* rats were also examined in the identical test battery to determine whether biparental inheritance of the truncating mutation alters behavioral outcomes. We found that *Magel2^m−/p−^* rats exhibited altered anxiety-like behaviors and sociability. In the elevated circle maze, *Magel2^m−/p−^* rats, in comparison to wild-type littermates, spent more time in the open regions (Fig. 3A) and less time in the closed regions (Fig. 3A), and transitioned more frequently between the two areas (Fig. S2A). In the three-chamber test for indirect social interaction, a repeated measures two-ANOVA indicated a significant genotype-by-sex interaction in three test parameters (Table S1). Post-hoc comparisons revealed that male *Magel2^m−/p−^* rats showed a comparable amount of time spent investigating a novel partner versus novel object, suggesting altered social approach behavior (Fig. 3B). Similar findings were observed in the number of events, or visits, to the partner versus object, as well as in the number of events to the chamber sides containing either the partner or object (Fig. S2B,C). These parameters were not altered in male or female wild-type littermates or female *Magel2^m−/p−^* rats, suggesting a male-specific social approach phenotype in rats homozygous mutant for the truncating *Magel2* mutation. To deepen our analysis of sociability, we used the juvenile dyadic direct social interaction test. Significant genotype-by-sex interactions followed by post-hoc analyses revealed a similar pattern of deficits in *Magel2^m−/p−^* rats. The time spent and number of events engaged in play-like behavior were significantly reduced in male *Magel2^m−/p−^* rats compared to wild-type littermates (Fig. 3C,D). Sniff-follow duration was normal (Fig. 3E); however, unlike other parameters, the number of events observed was significantly reduced in both male and female *Magel2^m−/p−^* rats compared to wild-type littermates (Fig. 3F). In contrast to rats heterozygous for the paternally inherited *Magel2* mutation, *Magel2^m−/p−^* rats did not display altered perseverative behaviors in either the marble-burying or block-chewing tests (Fig. 3G,H). Performance in the open-field test was normal (Fig. 3I; Fig. S2); therefore, the decreased anxiety-like behavior in the elevated circle maze and altered social behavior does not appear to be a result of alterations in gross locomotor function. Finally, in the evaluation of PPI of the acoustic startle response, we found that *Magel2^m−/p−^* rats displayed normal baseline startle reactivity and normal PPI (Fig. 3J,K).

### Paternal heterozygous or homozygous loss of *Magel2* in juvenile rats does not affect memory and learning performance or pain nociception

**Fig. 4.**
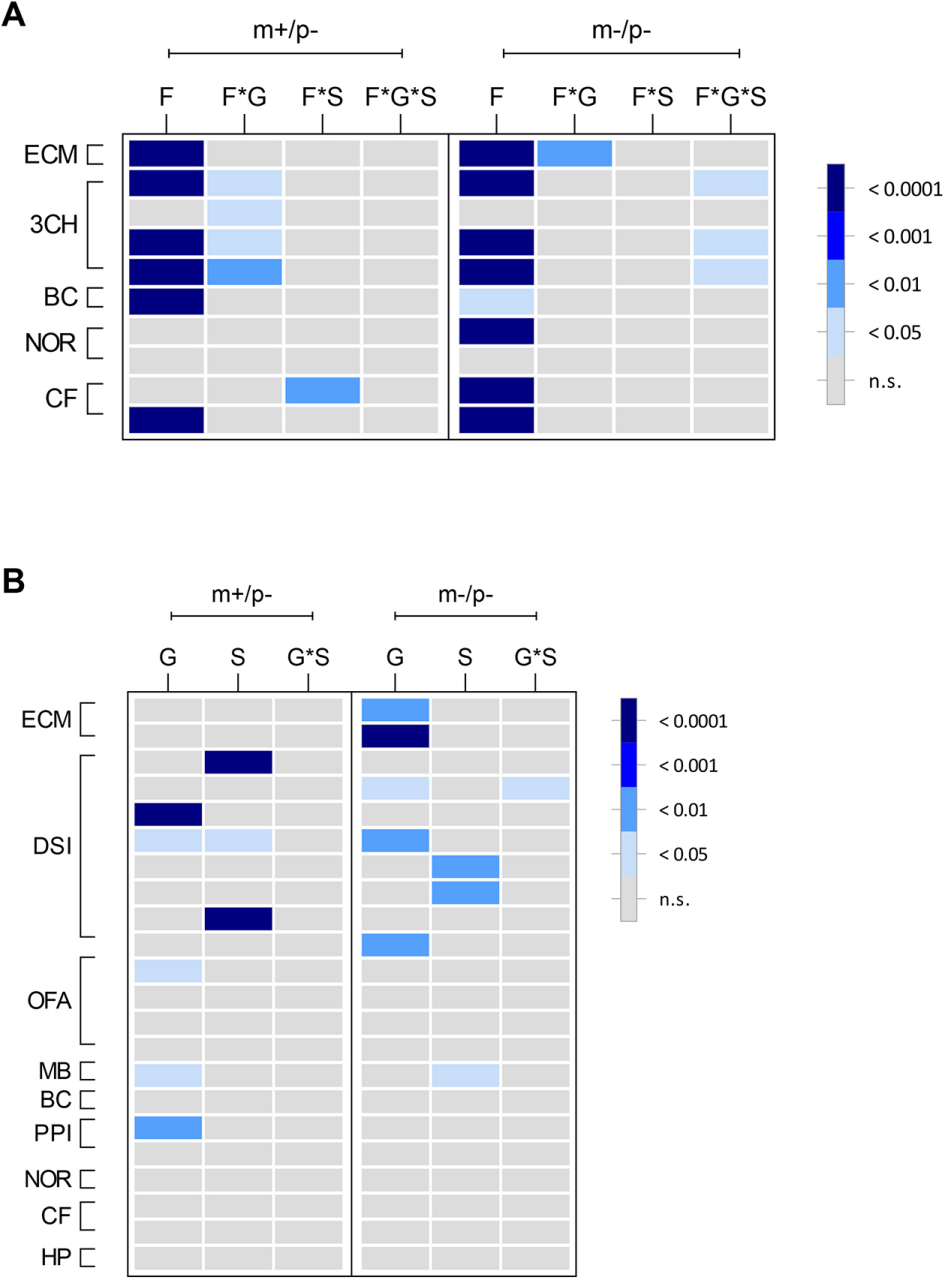
**Summary heatmap representation of mutant *Magel2* rat behavioral outcomes.** (A,B) Graphical representation showing the degree of statistical significance for both mutant *Magel2* lines relative to their wild-type littermate controls. Each row corresponds to a specific test parameter of the indicated procedure to the left outlined in brackets. Within each mutant line (shown along top of graph as m+/p− or m−/p−), each column represents the main effect or interaction for each statistical analysis. In each panel legend, the degree of statistical significance is indicated by relative gray to dark-blue color, each corresponding to a *P*-value ranging from not significant (n.s.) to *P*<0.0001. (A) For applicable procedures, repeated measures (RM) ANOVA was conducted for specific parameters of the elevated circle maze (ECM), three-chamber test (3CH), block-chew test (BC), novel object recognition test (NOR) and conditioned fear test (CF). F, factor; F*G, factor-by-genotype; F*S, factor-by-sex; F*G*S, factor-by-genotype-by-sex. (B) For parameters that were not analyzed by RM ANOVA, multivariate two-way ANOVA using genotype and sex as main effects followed by post-hoc analyses was conducted for ECM, 3CH, direct social interaction (DSI), open-field assay (OFA), marble burying test (MB), BC, acoustic startle and prepulse inhibition (PPI), NOR, CF and hot-plate test (HP). G, genotype; S, sex; G*S, genotype-by-sex. Specific parameters for each row in A and B are provided in Table S1.

SYS patients are frequently diagnosed with intellectual disability and developmental delay. To determine whether mutant rats for the truncating *Magel2* mutation exhibited deficits in learning and memory, *Magel2^m+/p−^* and *Magel2^m−/p−^* rats were tested on object recognition memory and aversive fear memory. Neither *Magel2^m+/p−^* nor *Magel2^m−/p−^* rats displayed observable deficits in the novel object recognition index (Fig. S3A,B) or in contextual or cue-based memory retrieval (Fig. S3C-F). As a relevant control measure for the mild-shock-based conditioned fear testing paradigm and to test whether alterations in pain sensitivity may be present in *Magel2* mutant rats, thermal pain reactivity to a 55°C heated surface was measured. No genotype differences were observed in the latency to first response in the hot-plate test (Fig. S3G,H). A heatmap (Fig. 4) summarizing the degree of statistical significance for behavioral outcomes identified in each *Magel2* mutant test cohort relative to their respective wild-type littermates is provided, analyzed by repeated measures two-way ANOVA (Fig. 4A) or multivariate two-way ANOVA (Fig. 4B). Corresponding statistical summary data are provided in Table S1.

### Body composition and weight are altered in paternal and homozygous *Magel2* mutant rats

**Fig. 5.**
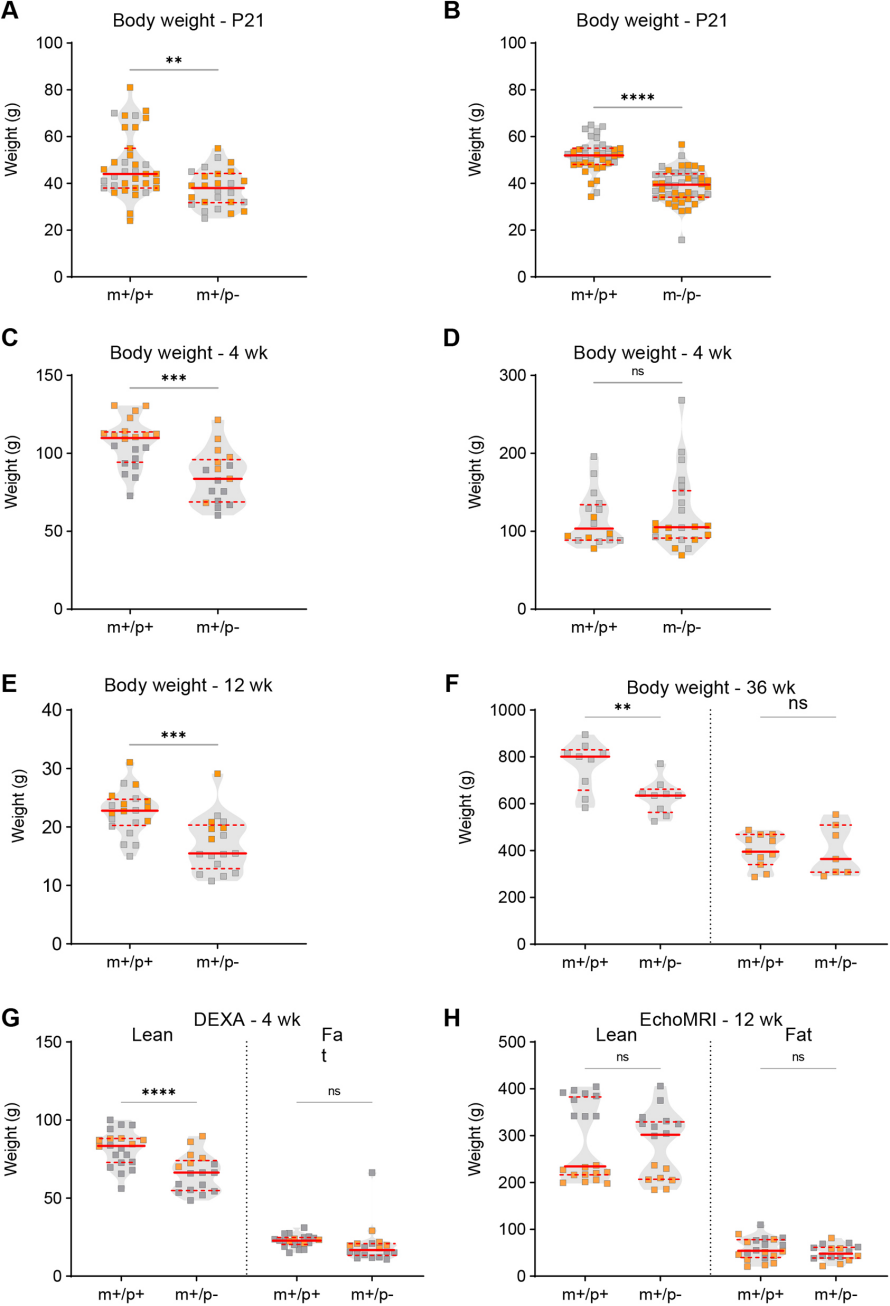
**Alterations in body weight and composition were found in rats with paternally inherited *Magel2* mutation.** (A-D) For *Magel2^m+/p−^* rats (m+/p−), *Magel2^m−/p−^* rats (m−/p−) and their respective wild-type littermates (m+/p+), body weight was assessed at P21 and at 4 weeks. At P21, both m+/p− and m−/p− rats showed a significant reduction in weight compared to that of their respective m+/p+ littermates (A,B); in contrast, at 4 weeks, m+/p− rats, but not m−/p− rats, showed a significant reduction in body weight (C,D). (E,F) *Magel2^m+/p−^* (m+/p−) rats showed decreased body weight at 12 weeks (E); at 36 weeks, only male m+/p− rats still showed reduced body weight (F). (G,H) Body composition measurements of lean and fat mass were evaluated by dual-energy x-ray absorptiometry (DEXA) at 4 weeks and magnetic resonance imaging (MRI) at 12 weeks. At 4 weeks, *Magel2^m+/p−^* (m+/p−) rats showed a significant reduction in lean, but not fat, mass (G); at 12 weeks, the absence of a genotype-by-sex interaction resulted in an overall genotype group analysis; no differences were observed between m+/p− and their respective m+/p+ littermates (H). Violin plots were used to indicate data density and distribution, with gray (males) and orange (females) squares within each genotype/group category; solid red lines indicate the median and dashed red lines indicate quartiles. **P<0.01; ***P<0.001; ****P<0.0001; ns, not significant; mutant rats relative to wild-type littermate controls; two-way ANOVA with genotype and sex as factors, followed by post-hoc analyses as appropriate.

**Fig. 6.**
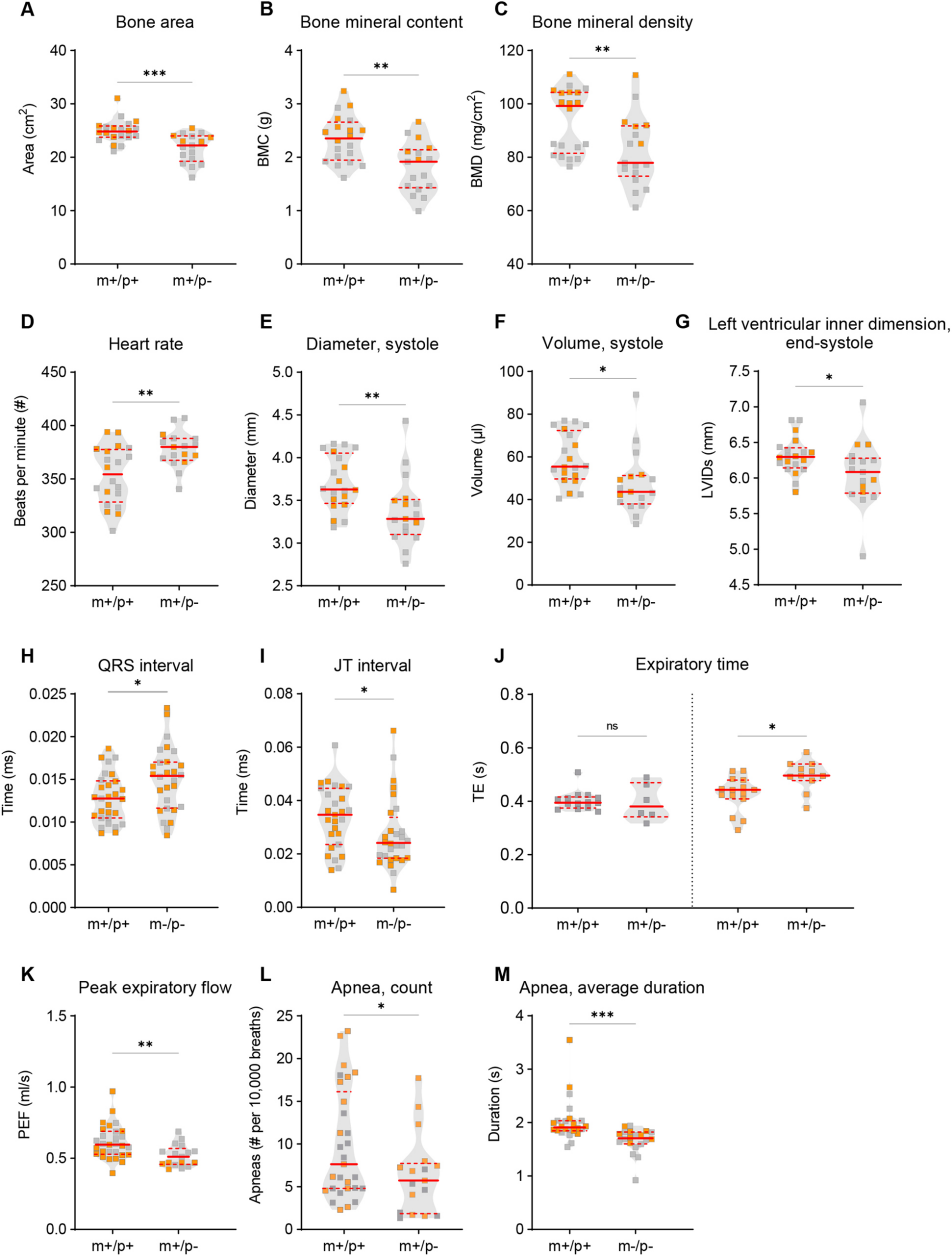
**Skeletal, cardiac and respiratory phenotypes in *Magel2* mutant rats.** (A-C) DEXA scans showed that *Magel2^m+/p−^* (m+/p−) rats, compared to their respective wild-type littermates (m+/p+), had a significant decrease in bone area (A), bone mineral content (BMC; B) and bone mineral density (BMD; C). (D-G) Cardiac morphology by echocardiogram analysis revealed a significant increase in heart rate (D), and decrease in ventricular diameter (E) and volume (F) during systole in m+/p− rats compared to m+/p+ littermate controls. End-systole left ventricular inner dimension during systole (LVIDs) was also reduced in m+/p− rats compared to m+/p+ littermate controls (G). (H,I) *Magel2^m−/p−^* (m−/p−) rats, compared to their respective wild-type littermates (m+/p+), showed altered cardiac electrical activity measured by electrocardiography; a significant increase in QRS interval (H) and decreased JT interval (I) was observed in m−/p− rats. (J-M) In unrestrained whole-body plethysmography, both m+/p− and m−/p− rats showed alterations in various breathing-related parameters. Although expiratory time (TE) was increased in female, but not male, m+/p− rats (J), genotype differences as a group were observed for peak expiratory flow (PEF; K). Decreased apnea count was observed in m+/p− rats (L); in contrast, the average duration of apneas was decreased in m−/p− rats (M). Violin plots were used to indicate data density and distribution, with gray (males) and orange (females) squares within each genotype/group category; solid red lines indicate the median and dashed red lines indicate quartiles. *P<0.05; **P<0.01; ***P<0.01; ns, not significant; multivariate two-way ANOVA using genotype and sex as main effects followed by post-hoc analyses were conducted when appropriate. A statistical summary of physiology data is provided in Table S2.

Individuals with *MAGEL2* variants exhibit several structural and functional alterations of peripheral organ systems; these features have been reported to co-occur with behavioral phenotypes (Patak et al., 2019). Therefore, to evaluate whether disruption of rat *Magel2* manifests physiological alterations that co-occur with behavioral phenotypes, we set out to test additional measurements of cardiac, skeletal and respiratory systems. Analyses of body morphological content, such as muscle and fat percentages, were performed to examine the tissue content and overall weight differences. Both *Magel2^m+/p−^* and *Magel2^m−/p−^* rats exhibited decreased total body weight in comparison to that of their wild-type littermates at postnatal day (P)21 (Fig. 5A,B). At 4 weeks of age, *Magel2^m+/p−^* rats continued to display decreased total weight compared to that of wild-type littermates, whereas *Magel2^m−/p−^* rats appeared to show no difference at the same age (Fig. 5C,D). Weight differences continued to persist at 12 weeks of age; however, at 36 weeks of age, a significant genotype-by-sex interaction followed by post-hoc analyses revealed that only male *Magel2^m+/p−^* rats showed a weight difference compared to their respective male wild-type littermates (Fig. 5E,F). Evaluation of lean and fat mass at 4 weeks of age identified a decrease in lean, but not fat, mass at 4 weeks of age in *Magel2^m+/p−^* rats; a similar analysis of lean and fat mass at 12 weeks of age did not reveal any significant group differences (Fig. 5G,H). In addition to altered total body weight and lean weight, *Magel2^m+/p−^* rats displayed significantly decreased bone area, mineral content and density at 4 weeks of age (Fig. 6A-C).

### Select cardiac structural and functional dysfunction is observed in *Magel2* mutant rats

Cardiac function was evaluated through echocardiography and electrocardiography tests, which measure the functionality and electrical properties of the heart. To examine the health of cardiovascular systems of *Magel2^m+/p−^* rats, we performed echocardiograms at a 1 month time point. A rodent echocardiogram enables ultrasound-based detection of the chambers of a heart, which can then be analyzed for potential abnormalities (Liu and Rigel, 2009). *Magel2^m+/p−^* rats displayed an increased heart rate (Fig. 6D), concomitant with systolic deficiencies defined as the phase of heart muscle contraction to pump blood to the body. These impairments included decreased diameter and reduced volume during systole (Fig. 6E,F) and decreased diameter of the left ventricle during end-systole (Fig. 6G). In contrast, although *Magel2^m−/p−^* rats were normal across structural parameters, several phenotypes were observed by electrocardiography, including elevated QRS interval (Fig. 6H), which corresponds to the time taken from the initial depolarization of the ventricle to the repolarization for the next contraction, and decreased JT interval (Fig. 6I), which corresponds to a decrease in time taken to ventricular repolarization.

### Paternal and homozygous mutant *Magel2* mutant rats have altered respiration, including altered apnea-based measurements

**Fig. 7.**
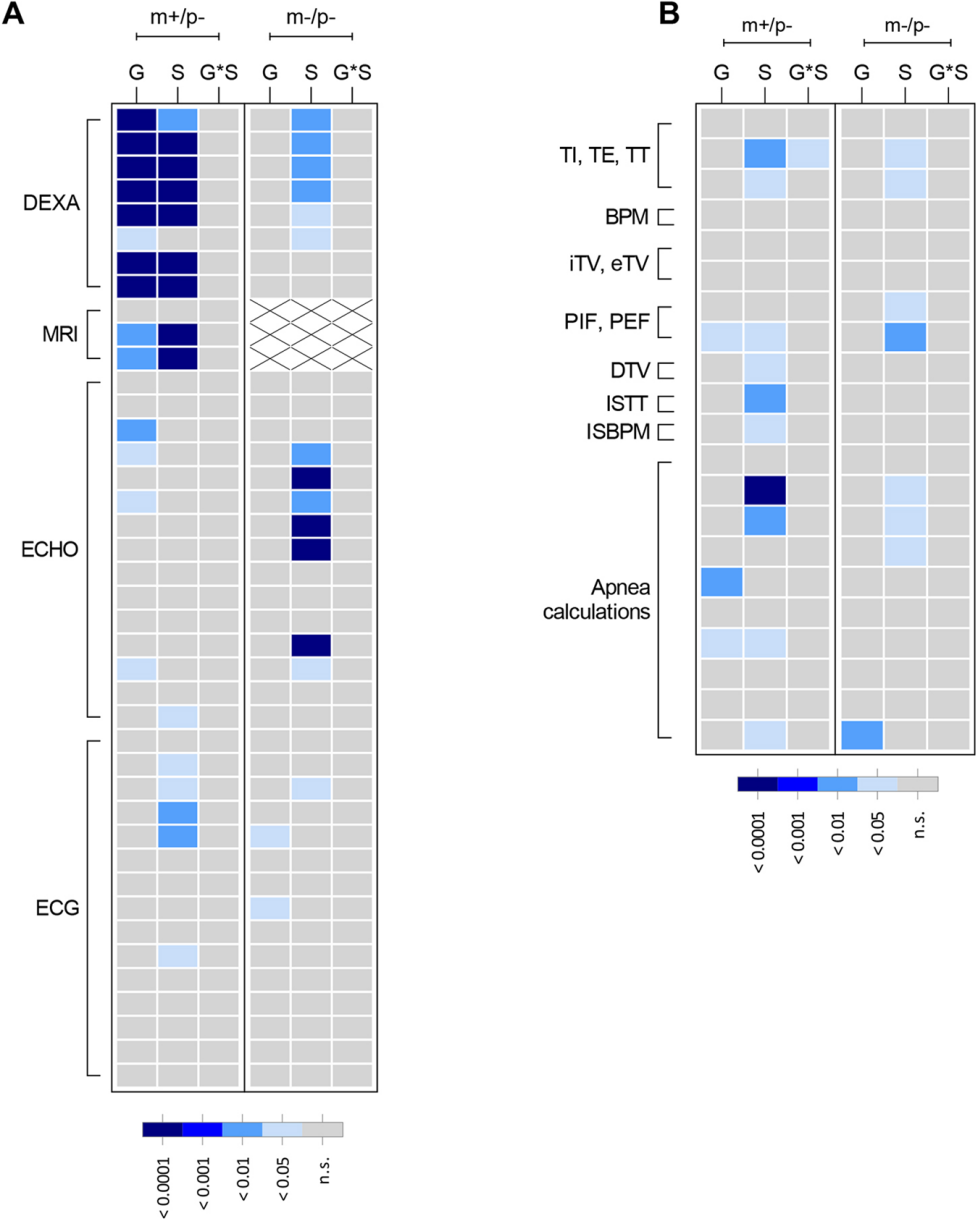
**Summary heatmap representation of mutant *Magel2* rat physiology and plethysmography outcomes.** Graphical representation showing an overview of the degree of statistical significance for both mutant *Magel2* lines studied relative to their wild-type littermate controls. Each row corresponds to a specific test parameter of the indicated procedure to the left outlined in brackets. Within each mutant line studies (shown along top of graph as m+/p− or m−/p−), each column represents the main effect or interaction for each statistical analysis. As shown in each panel legend, the degree of statistical significance is indicated by relative gray to dark-blue color, with each color corresponding to a *P*-value ranging from not significant (ns) to *P*<0.0001. For MRI, white boxes with ‘×’ indicate not measured. (A) Multivariate two-way ANOVAs with post-hoc analyses were conducted and graphically displayed for dual-energy x-ray absorptiometry (DEXA), quantitative magnetic resonance technology (MRI), echocardiogram (ECHO) and electrocardiography (ECG). (B) Multivariate two-way ANOVAs with post-hoc analyses were conducted and graphically displayed for parameters collected during unrestrained whole-body plethysmography. Parameters displayed are inspiratory time (TI), expiratory time (TE), total breath time (TT), breaths per minute (BPM), inspiratory tidal volume (iTV), expiratory tidal volume (eTV), peak inspiratory flow (PIF), peak expiratory flow (PEF), tidal volume (DTV), ratio TT change (ISTT) and ratio BPM change (ISBPM). Parameters for each row in A and B are provided in Table S2. G, genotype; S, sex; G*S, genotype-by-sex.

It has recently been reported that up to 71% of patients with SYS experience some form of respiratory distress (McCarthy et al., 2018). Owing to this increased prevalence of respiratory features in SYS, *Magel2* rats were also examined for respiratory performance using unrestrained whole-body plethysmography. In comparison to wild-type littermates, *Magel2^m+/p−^* rats exhibited increased expiratory time, impacting peak expiratory flow (Fig. 6J,K). Furthermore, both *Magel2^m+/p−^* and *Magel2^m−/p−^* rats exhibited decreased apnea counts and duration (Fig. 6L,M). A heatmap (Fig. 7) summarizing the degree of statistical significance for organ physiology outcomes identified in each *Magel2* mutant test cohort relative to those of their respective wild-type littermates is provided, analyzed by multivariate two-way ANOVA for body composition and cardiac measurements (Fig. 7A) and for breathing parameters (Fig. 7B). Corresponding statistical summary data are provided in Table S2.

### Detection of wild-type and truncated rat MAGEL2 peptides by mass spectrometry

Detecting endogenous MAGEL2 through antibody-based approaches has been notoriously difficult. Cellular studies have successfully visualized modified MAGEL2 species through genetic approaches such as GFP and Myc labeling (Hao et al., 2013). Recently, a polyclonal antibody was generated against the C-terminal of MAGEL2 and was successful in delineating the discrepancy between wild-type and *LacZ* knock-in *Magel2* mice, as well as in SYS cell lines (Chen et al., 2020b). However, this antibody would be unable to identify whether a truncated MAGEL2 persists in either our rat model or in SYS patient cell culture, as the N-terminus is predicted to be absent in truncating mutations.

**Fig. 8.**
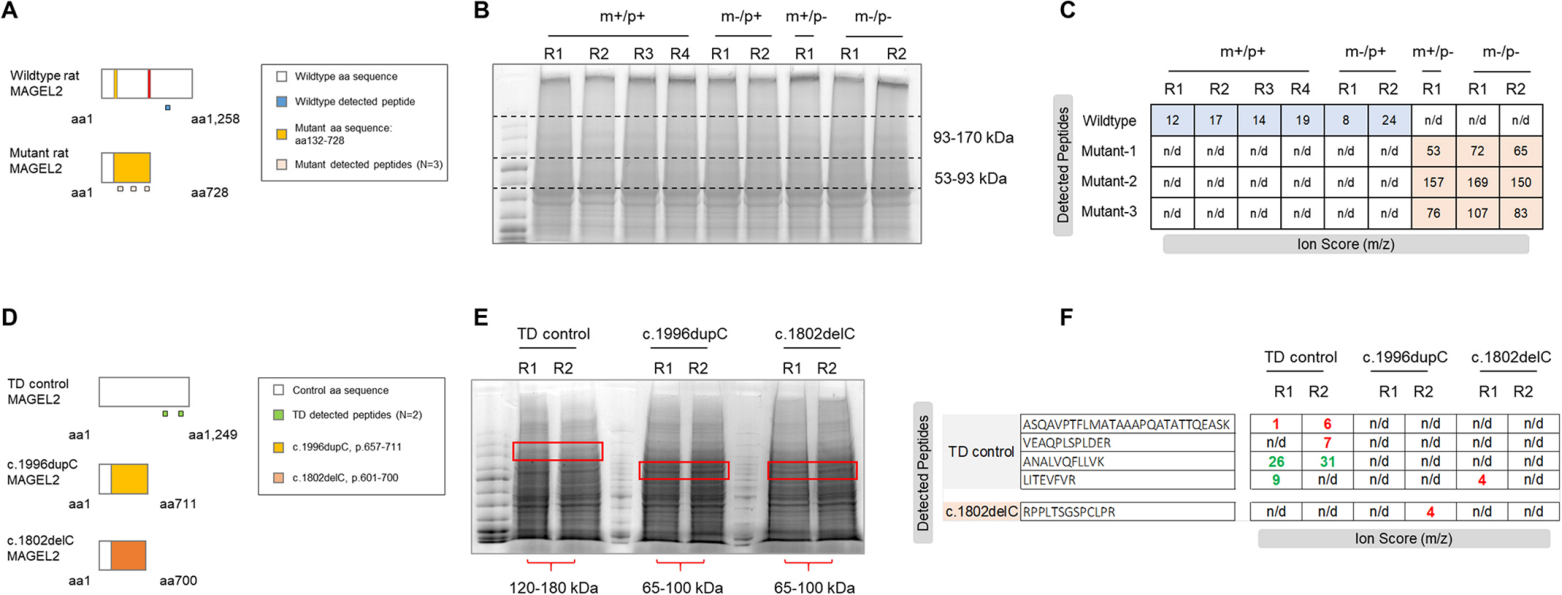
**Detection of MAGEL2 peptides in *Magel2* mutant rats and Schaaf-Yang syndrome (SYS) human induced pluripotent stem cell (hiPSC) lines.** (A) Schematic diagram showing the expected full-length wild-type MAGEL2 protein compared to the truncated mutant MAGEL2 protein. Within each schematic, the white box indicates wild-type amino acid (aa) sequence; the yellow box indicates the mutant aa sequence. The anticipated detection of wild-type (blue boxes) and mutant (light-pink boxes) peptides are shown below each schematic diagram. (B) Representative image of SDS-PAGE gel containing wild-type (m+/p+), maternally inherited (m−/p+), paternally inherited (m+/p−) and homozygous *Magel2* (m−/p−) hypothalamus samples with excisions from 93-170 kDa and 53-93 kDa. R corresponds to each biological replicate. (C) Ion scores (m/z) of detected wild-type and mutant peptides within each allelic combination. R corresponds to each biological replicate. Rat wild-type MAGEL2 aa sequence corresponding to *n*=1 wild-type peptide was detected in m+/p+ and m−/p+, and not detected in m+/p− or m−/p−, rat hypothalami. All three predicted mutant peptides were only detected within the novel region (p.132-728) in m+/p− and m−/p− rat hypothalami. n/d, not detected. (D) Schematic diagram showing expected full-length MAGEL2 protein in typical developing (TD) control hiPSC lines compared to predicted truncated mutant MAGEL2 protein in either c.1996dupC or c.1802delC hiPSC lines derived from SYS individuals. Boxes indicate wild-type aa sequence (white), mutant aa sequence for c.1996dupC (yellow), mutant aa sequence for c.1802delC (orange) and anticipated TD-detected peptides (green). (E) Representative image of SDS-PAGE gel containing TD control, c.1996dupC and c.1802delC samples. R indicates biological replicate number. Red boxes outline the excisions from 120-180 kDa (TD hiPSCs) and 65-100 kDa (SYS hiPSCs). (F) Ion scores of detected TD control and mutant peptides within each sample replicate. not detected (n/d); green, strong/confident peptide detection; red, poor/low confident peptide detection. Both TD control replicates had detectable MAGEL2 peptides; c.1996dupC and c.1802delC replicates did not display detectable control or mutant sequences.

To determine the presence or absence of wild-type MAGEL2 in our rat model, we utilized high-performance liquid chromatography mass spectrometry. Hypothalamic brain samples from *Magel2^m+/p−^*, *Magel2^m−/p+^*, *Magel2^m−/p−^* and wild-type littermates were run on an SDS-PAGE gel, and a region between 93-170 kDa was excised (wild-type rat MAGEL2 predicted molecular mass is 132 kDa), gel purified, trypsin digested and analyzed by mass spectrometry in an attempt to identify the full MAGEL2, a truncated species, or if no protein was present across all genotypic combinations (Fig. 8). The 8 bp deletion generated a frameshift event, p.Ser132Glys, which is predicted to prematurely terminate at amino acid 843, possibly yielding a 71.5 kDa truncation product (Fig. 8A). The most strongly detected peptide sequence from all wild-type littermate samples derived from paternal, maternal and homozygous inheritance mating schemes was NLPASSETFPATSR. This digested peptide is a translation of nts (c.2725_2766, NC_005100.4), which is 1982 bp downstream of the 8 bp deletion and 188 bp after the mutant premature stop codon, thus only detectable from a wild-type allele. Ion scores for the wild-type peptide from each sample were compared, and indicate that the wild-type peptide was not detectable in *Magel2^m+/p−^* or *Magel2^m−/p−^* hypothalamic samples but was reliably detected in *Magel2^m−/p+^* samples (Fig. 8A,B). This suggests that (1) a full MAGEL2 translation product is very unlikely in any of the mutant states (leakiness is not occurring from the imprinted maternal allele) and (2) maternal heterozygous animals have detectable wild-type peptides. However, a truncated protein could not be empirically discounted. To search for the existence of a truncated MAGEL2 product, the mutant *Magel2* sequence was used to generate a list of possible trypsin-digested peptide products, and a region between 53-93 kDa on an SDS-PAGE gel was excised. Three mutant peptides, aligning between the 8 bp deletion and the premature stop codon, were detected in only *Magel2^m+/p−^* and *Magel2^m−/p−^* samples with high ion scores (Fig. 8C). The mutant peptides were not detected in any wild-type samples and did not align to the translated wild-type *Magel2* sequence. Samples from *Magel2^m−/p+^* hypothalamus did not yield detectable mutant peptide sequences, complementing our previous cDNA sequencing data (Fig. 1) and further demonstrating that rat *Magel2* is paternally expressed. We report, to our knowledge, the first protein-based detection of endogenous and unmodified wild-type MAGEL2, along with evidence suggesting the presence of a truncated MAGEL2 species resulting from the *Magel2* mutation in paternal and homozygous mutant rat hypothalami.

### Rat and SYS predicted truncated MAGEL2 are similar in size, but human peptides are not detectable in SYS human induced pluripotent stem cell (hiPSC) protein lysates

Mass spectrometry results were particularly interesting in relation to SYS, as SYS patients are predicted to have truncating mutations in *MAGEL2* (Schaaf et al., 2013). We asked whether the truncated protein generated in our rat model was comparable to those predicted in SYS. An extensive report of SYS patients revealed a ‘mutational hotspot’ in *MAGEL2* from c.1990 to c.1996, a region found in the majority of molecularly confirmed cases of SYS (McCarthy et al., 2018). Sixty-one of the 78 reported cases had the mutation c.1996dupC. This prolific mutation is predicted to generate a predicted truncated protein of 72 kDa terminating before the USP7-binding domain (U7B) and MAGE homology domain (MHD). In comparison, the 71.5 kDa mutant rat MAGEL2 truncated protein is nearly identical in size and terminates before its predicted U7B and MHD regions.

To determine whether predicted truncating mutations in human *MAGEL2* result in a truncated protein, we examined hiPSC SYS cell lines and typical developing (TD) controls using targeted mass spectrometry. A similar approach to distinguishing wild-type and mutant rat MAGEL2 peptides was utilized for the human MAGEL2 sequence (Fig. 8D). The SYS hiPSC samples contained either the c.1996dupC or the c.1802delC frameshift mutations. TD and SYS protein lysates were SDS-PAGE separated, followed by gel excision of regions predicted for either wild-type or mutant MAGEL2 protein sizes (Fig. 8D,E). The c.1996dupC is predicted to result in a mutant peptide sequence beginning at amino acid 666 through its termination at 711, whereas the c.1802delC frameshift occurs at amino acid 601 and terminates at 700 (Fig. 8D,E). Mass spectrometry results indicated that wild-type human MAGEL2 peptides were detectable in the TD controls samples, but neither wild-type peptides prior to the frameshift mutations nor mutant sequences were detected in either of the SYS hiPSC lines (Fig. 8F).

## DISCUSSION

Utilizing the TALEN approach (Meek et al., 2017), an 8 bp deletion was generated in the single coding exon of rat *Magel2*, resulting in a frameshift event followed by a downstream premature termination codon. This mutation was predicted to generate a truncated MAGEL2, separating itself from murine models harboring large gene deletions (Kozlov et al., 2007; Schaller et al., 2010). By generating a truncating *Magel2* mutation in a model with unique experimental advantages, such as the laboratory rat, our study has revealed several common and distinct murine alterations in neurobehavioral outcomes and functional changes in organ physiology. F1 hybrid and Sanger sequencing studies confirmed paternal transmission and expression of rat *Magel2*, providing additional confidence in a conserved regulatory mechanism of expression across rat, human and mouse loci (Boccaccio et al., 1999) in typical developing animals. Although Sanger sequencing of the endpoint PCR product across the 8 bp deletion in the *Magel2* rat model did not reveal maternal ‘leaky’ expression, full gene deletions in mice have been reported to release the maternal copy of *Magel2* and other imprinted loci to a limited extent (Matarazzo and Muscatelli, 2013; Rieusset et al., 2013). Future studies examining the methylation of the maternal allele could further elucidate genomic imprinting in the rat, and whether truncating mutations result in low-level maternal expression undetectable in this current study.

At the transcript and protein levels, our analyses of hypothalamic tissue and hiPSCs were instructive. Unlike full *Magel2* deletion models, which lack endogenous *Magel2* transcript, normal levels of mRNA abundance were observed across all *Magel2* genotypes regardless of uniparental and biparental contribution. This was unsurprising as single-exon genes are not usually susceptible to nonsense-mediated decay and demonstrated that differential inheritance of the mutation did not impact the expected, imprinted expression pattern of the gene. This observation has previously been reported in other monogenic rat models with normal levels of transcript in the presence of altered protein expression (Veeraragavan et al., 2016). To our surprise, by mass spectrometry, both wild-type and mutant-derived peptides were detected from rat hypothalamic, but not SYS hiPSC, protein samples. To our knowledge, this is the first reported attempt to detect truncated MAGEL2 peptides in SYS hiPSCs as patient mutations have only been evaluated by DNA sequencing. Several possibilities may explain these results compared to the observations in rat: (1) mutant MAGEL2 levels may be extremely low, making it difficult to detect via mass spectrometry; (2) the samples were not neuronal cultures (only hiPSCs), whereas *MAGEL2* is neuronally enriched; and (3) the mutant protein may naturally undergo degradation and is not present actively in these samples. Future studies examining the extent of MAGEL2 presence in SYS samples could provide deeper insight into the cellular consequences of this truncated protein. Although truncated MAGEL2 protein has still only been predicted to exist in patients with SYS, it is possible that our rat model using hypothalamic brain samples can be used as a model to study truncated MAGEL2 as predicted in SYS. These results may further support the hypothesis of a neomorphic truncated mutant MAGEL2, which may explain the observed clinical severity separating SYS from full *MAGEL2* PWS deletion.

A primary goal of the comprehensive phenotypic analyses was to evaluate for phenotypic consequences in rats with a truncating *Magel2*. Most literature on *MAGEL2*-related disorders is derived from young children (Patak et al., 2019), with limited studies of the adult phenotype in SYS (Marbach et al., 2020). Our neurobehavioral analysis focused on a neurologically comparable rodent age of 3-6 weeks (Semple et al., 2013). Deploying a thorough and comprehensive behavioral battery across multiple domains, we found that *Magel2^m+/p−^* and *Magel2^m−/p−^* rats displayed several alterations from wild-type littermates. *Magel2^m+/p−^* rats exhibited reductions in marble burying during a marble interaction test, suggesting anxiety-like features reflective of a neophobic, avoidant response (Thomas et al., 2009). These data are further strengthened by findings across *Magel2^m+/p−^* and *Magel2^m−/p−^* rats. Although *Magel2^m+/p−^* rats appeared to have a non-significant reduction in anxiety-like behavior measured in the elevated circle maze, *Magel2^m−/p−^* rats clearly displayed increased time in the open regions of the elevated circle maze. In addition, the play-specific sociability phenotypes observed in the *Magel2* rats point to disruption of systems for social processes that may govern direct interaction with respect to time and frequency of social engagement. Social processes in individuals with MAGEL2-related disorders are disrupted, as ASD and social deficits are frequently reported (Patak et al., 2019); reduced sociability has also been reported in individuals with PWS compared to individuals with Down syndrome (Rosner et al., 2004). These results appear to indicate that *MAGEL2* is responsible for select systems within the social processes domain. Finally, although PPI was normal across all genotypes, *Magel2^m+/p−^* rats showed a statistically significant reduction in the startle response. *Magel2^m−/p−^* rats, in contrast, did not show statistically significant differences in either startle reactivity or PPI, although it is worth noting that a trend in reduced startle reactivity was observed. Based on the startle circuit, the concordance MAGEL2 disruption may be specifically tied to long-range projections that connect across brain regions responsible for PPI and startle reactivity.

Changes observed across these behavioral domains may be reflective of abnormal production and/or processing of the neuropeptide oxytocin (OT; also known as OXT). OT is derived within the hypothalamus, the region in which *Magel2* displays its greatest enrichment (Hao et al., 2015), and acts as a neuromodulator in the brain regions that regulate the disrupted behaviors of the *Magel2* rat (Mitre et al., 2018). Interestingly, previous studies using *Magel2*-deficient mouse models have discovered similar changes in behavioral phenotypes along with molecular alterations of the OT system, some which were correctable following early OT administration (Schaller et al., 2010; Meziane et al., 2015; Reichova et al., 2021; Bertoni et al., 2021). Future analysis of neuronal subtypes regulating OT is warranted, as alterations in activity and outgrowth have also been reported (Ates et al., 2019; Reichova et al., 2021). *MAGEL2* interacts with the WASH complex to facilitate intracellular protein trafficking (Hao et al., 2013), and *Magel2*, when disrupted, has resulted in significant reductions in OT within the hypothalamus of mice (Chen et al., 2020b). Examining the OT system and changes within intracellular signaling pathways in truncated *Magel2* rats may further elucidate the changes that may be occurring in SYS.

Although different individual sets of phenotypes were uncovered between *Magel2^m+/p−^* and *Magel2^m−/p−^* rats, examination from the perspective of broader phenotypic changes (Figs 4 and 7) provides additional insight into how *Magel2* dysfunction in the rat may modulate specific domains, especially within the framework of Research Domain Criteria (RDoC) (Insel et al., 2010). Within this perspective of categorical alterations to units of analyses, the results from the *Magel2* mutant rat model implicate altered negative valence behaviors (anxiety-like and perseverative behaviors represented by measures derived from marble burying and elevated circle maze) and altered social processes (represented by measures derived from three-chamber and direct social interactions tests). This interpretation is also consistent with findings from paternal mutant mice reported to display reduced levels of anxiety-like behaviors in an elevated plus maze and alterations in sociability (Fountain et al., 2017; Meziane et al., 2015). Endophenotypic change within the negative valence domain, such as changes in exploration of an elevated circle maze and marble burying, are generalized as modulating compulsive/anxiety-like behaviors (Kremer et al., 2021). That people with PWS (Goldstone, 2004; Skokauskas et al., 2012) and SYS (Marbach et al., 2020; McCarthy et al., 2018) display changes in negative valence, as rates of anxiety and compulsive behaviors are increased compared to those in people with non-PWS intellectual disabilities, further strengthens the interpretation of these data from an RDoC-based framework. The source of variation between datasets may occur during embryonic development from the maternal allele in *Magel2^m−/p−^* rats. Some imprinted genes switch between bi- and mono-allelic expression during this period (SanMiguel and Bartolomei, 2018), possibly allowing for excess truncated MAGEL2 contribution during development in the homozygous genotype. Future embryonic studies examining the expression of the truncated *Magel2* may shed light on this genetic possibility and explain the phenotypic differences observed.

The second purpose of this study was to evaluate the organ physiology and potential consequences that may arise from a truncating rat *Magel2* mutation. Many individuals with MAGEL2-related disorders are afflicted with debilitating and disruptive changes outside the CNS. The rat is positioned to be an excellent tool for peripheral physiology studies, given its size and large tissue area, and a preferred system for therapeutic validation (Wöhr and Scattoni, 2013). From our studies of body composition, cardiac function and structure, and respiration, we identified several co-occurring features due to the loss of MAGEL2 in the rat. First, disruption of *Magel2* in rats leads to alterations in body composition, highlighted by reduced body weight, whereas gross obesity was never observed. However, findings from *Magel2* mouse models have identified similar metabolic phenotypes in fat mass and body weight, but also have induced obesity, although via a high-fat diet (Arble et al., 2016; Bischof et al., 2007). These rats were maintained on a standard rodent chow, so the possibility remains that rats on a high-fat diet may yield similar results to mouse models. It is worth noting that people with truncating *MAGEL2* variants do not display the hallmark PWS hyperphagia-induced obesity (Chen et al., 2020a; McCarthy et al., 2018). However, a majority of cases within a small cohort of adult patients (*n*=5 of 7) were clinically obese with food-seeking behaviors, as reported by caregivers (Marbach et al., 2020). Taken together, the murine model findings strengthen the notion that disruption in *Magel2* across species results in alterations in body composition. Second, through echocardiography, enabling examination of the left ventricle of the heart (Liu and Rigel, 2009), *Magel2^m+/p−^* animals exhibited elevated heart rates and systolic dysfunction. These bidirectional cardiac changes may be a compensatory mechanism to maintain normal blood flow, as the ejection fraction in these animals was normal. A possible explanation for these dual observations could be a compensation mechanism; to maintain regular blood output with a smaller left ventricular diameter and mass, the heart might compensate by increasing the frequency of contractions. Further, *Magel2^m−/p−^* rats, when examined by electrocardiography, enabling the detection of electrical activity of the heart, displayed an elongated QRS interval, suggesting that the time to depolarize the heart was altered. Increase in the QRS interval has been attributed to bradycardia (reduced heart rate) (Da Costa et al., 2002). Additionally, the JT interval, a normative measurement of ventricular repolarization, was reduced in *Magel2^m−/p−^* rats. Together, these findings suggest that electrical heart activity is modulated without affecting the survival of the animals. Relevant to SYS, fatal cases of congenital heart disease have been reported in *MAGEL2* patients (Chen et al., 2020a), making these cardiac findings especially relevant. Third, the finding that *Magel2* mutant rats experience altered apnea metrics compared to wild-type littermates is interesting when considering elevated rates of obstructive sleep apnea in SYS (Powell et al., 2020). Although respiration was measured in the unrestrained, whole animal during periods of rest to ensure that the breathing measurements were not perturbed by movement, further studies investigating the convergence of sleep behaviors and respiratory performance in these rats would be instructive on breathing abnormalities as surrogate translational markers for SYS and MAGEL2-related disorders.

In conclusion, the results reported here demonstrate that the laboratory rat is a viable model with which to study the molecular, behavioral and organ function implications resulting from a truncating mutation in *Magel2*. The conservation of genomic imprinting of the *Magel2* gene across rat and human allows for another preclinical tool in the effort to provide future therapies to a growing patient population. Given the nature of the genetic manipulations in mouse models of total *Magel2* deficiency, the availability of a truncated rat MAGEL2 protein could provide valuable additional insight into potential molecular discrepancies that exist between the full deletion and truncating genotypes. Preclinical studies on gene-based interventions have demonstrated the promise of targeted approaches for modulating gene levels, especially for imprinting disorders such as Angelman syndrome (Meng et al., 2015; Wolter et al., 2020); however, in the case of truncating mutations in *MAGEL2*, future gene-based therapy efforts will need to take careful considerations when attempting to activate the silent maternal copy along with possibly targeting the existing pathogenic truncated MAGEL2 species through techniques such as proteolysis-targeting chimeras (Schapira et al., 2019). The current rat model may serve as a useful benchmark along with full gene deletion mouse models for identifying the best approaches for wild-type restoration and reduction of the mutant protein. Overall, these data on the rat model, and those reported in mice, link the loss of wild-type MAGEL2 to phenotypes consistent with altered negative valence and social processes, and consistent with alterations observed in people with MAGEL2-related disorders.

## MATERIALS AND METHODS

### Animals

All animal experiments were carried out in accordance with research protocol AN-1695 and approved by the Baylor College of Medicine Institutional Animal Care and Use Committee. The Animal Welfare Assurance at Baylor College of Medicine is approved by the National Institutes of Health (NIH) Office of Laboratory Animal Welfare and meets the requirements of the Public Health Service Policy on Humane Care and Use of Laboratory Animals, assurance number D16-00475 (A3823-01). Rats were housed in a temperature-controlled environment on a 12 h light:12 h dark cycle with *ad libidum* access to food and water. The *Magel2* mutation was created by TALEN technology, which generated an 8 bp deletion, c.735-742del, resulting in a frameshift event followed by an early termination site, p.Ser132GlysfsTer728. All rats in this study were bred on a SD background (Crl:CD-IGS background, strain code 001, Charles River Laboratories). Paternally inherited *Magel2* heterozygous rats (*Magel2^m+/p−^*, also denoted as m+/p−) and wild-type littermates (*Magel2^m+/p+^*, also denoted as m+/p+) were generated by mating wild-type females to males heterozygous for the *Magel2* mutation. Homozygous animals (*Magel2^m−/p−^*, also denoted as m−/p−) for the *Magel2* mutation were generated by mating heterozygote females with heterozygote males. Maternally inherited *Magel2* heterozygous rats (*Magel2^m−/p+^*, also denoted as m−/p+) were generated by mating heterozygous females to wild-type males. At P14, ear punch biopsies were used for rat identification and PCR genotyping with the following primers: forward, 5′-CAGATGGCCCAATCTTCAAC-3′; reverse, 5′-TCCCAGGAGGGTGTGTCATA-3′. PCR products were separated by 4% agarose gel electrophoresis, stained and visualized using an automated gel imaging system (Gel Doc XR+, Bio-Rad). The expected-size products for the wild-type allele (115 bp) and mutant allele (107 bp) were used for genotype interpretation. At P21, rats were weaned and randomly assigned to housing configurations of two to three rats per cage. Wild-type animals used in the three-chamber approach and direct social interaction assays were bred from a separate set of mating cages, generating conspecific non-littermate partners.

### qRT-PCR

Animals were anesthetized via isoflurane induction, and hypothalami were manually dissected from brain samples and flash frozen in liquid nitrogen. RNA was extracted from dissected specimen using TRIzol reagent (Thermo Fisher Scientific). One to two micrograms of total RNA were used for cDNA synthesis; mRNA was selectively reversed transcribed by targeting the poly(A) tail region using only Oligo(dT)_20_ primers (SuperScript IV First-Strand Synthesis System, Thermo Fisher Scientific). SsoAdvanced Universal SYBR Green Supermix (Bio-Rad) was used for qRT-PCR with commercially available rat *Magel2* and *Gapdh* primers (Bio-Rad; PrimePCR^TM^ SYBR Green assay ID qRnoCED0053164 and qRnoCID0057018, respectively). Expression levels from qRT-PCR experiments utilizing *Magel2* mutant samples were processed as above and normalized to wild-type littermate levels. Statistical analysis of qRT-PCR data was performed using a one-way ANOVA with multiple comparisons with genotype as the main factor and graphically presented using GraphPad Prism (Version 9.4).

### Endpoint PCR and Sanger sequencing analysis

To determine whether *Magel2* in the rat exhibited parent-of-origin effects, wild-type SD (Crl:CD-IGS) females and LE (Crl:LE) males were bred to produce F1 hybrid offspring. Hypothalami from parental rats and offspring were dissected for RNA extraction and cDNA synthesis as described above. Endpoint PCR amplification was performed using the following primers: forward, 5′-TCTAGGGGCCCCAATGGT-3′; reverse, 5′-CTGCTGGGCTATCGGTGT-3′. The expected-size PCR product of 460 bp was gel extracted and purified for sequencing analysis (QIAquick Gel Extraction Kit, Qiagen). Sanger sequencing was conducted by the Baylor College of Medicine DNA Sequencing Core using a 3130XL Genetic Analyzer (Applied Biosystems). Alignment of chromatogram traces to analyze nt position 864 as either G or A was used to determine parent-of-origin inheritance and expression in typically developing rats through the paternal lineage in F1 hybrid test progeny. To confirm the paternal pattern of inheritance and expression in the TALEN-induced *Magel2* rat model, test progeny with uniparental heterozygous (either paternal or maternal heterozygous) and biparental homozygous inheritance of the *Magel2* mutation were generated. Hypothalami from test progeny were dissected for RNA extraction and cDNA synthesis as described above. Endpoint PCR amplification was performed using the genotyping primers that span the 8 bp deletion, and the PCR product was gel extracted and purified for Sanger sequencing. Presence of wild-type sequence in the chromatogram traces through the *Magel2* mutation site was interpreted as wild-type expression; presence of a gap in the sequence trace when aligned to a reference sequence was interpreted as expression of the mutated allele. Rat images in Fig. 1 were created with BioRender.com.

### Overview of behavioral procedures and statistical analyses of behavioral data

Behavioral performance of juvenile animals from 3 to 6 weeks of age was measured using several procedural tests similar to those used in previous studies (Veeraragavan et al., 2016), including anxiety-like behavior, indirect social approach, direct social interaction, locomotor activity, sensorimotor gating, perseverative behaviors, aversive fear memory and object recognition memory, and thermal nociception. Studies were conducted between the hours of 12:00 and 18:00 [Zietgeber time (ZT), ZT5-11] during the light cycle. Prior to commencing the test battery, each subject was handled for two consecutive days by a trained experimenter blinded to genotype. All assays were performed under dim lighting conditions (8-10 lux) and white noise (60-62 dB) unless otherwise stated. Subjects were habituated to the procedure room for 30 min prior to testing. Both sexes were examined in each behavioral cohort with male rats tested before female rats in each assay. Group sizes were calculated using a power of 0.8 (α=0.05) to detect a difference of one standard deviation based on subjects per genotype per sex. For the behavioral evaluation of paternally inherited *Magel2^m+/p−^* rat studies, *n*=12-19 rats per genotype per group were tested. For the behavioral evaluation of homozygous *Magel2^m−/p−^* rat studies, *n*=14-22 rats per genotype per group were tested. All data were analyzed and represented as individual units within the violin plot-formatted graphs. The experimental design strategy deployed for our studies adheres to Animal Research: Reporting of In Vivo Experiments (ARRIVE) guidelines (Percie du Sert et al., 2020), follows reported study recommendations (Landis et al., 2012), and has been used for the analysis of genetic rat models by our group and others (Berg et al., 2020; Hamilton et al., 2014; Ku et al., 2016; Veeraragavan et al., 2016). Post-hoc Tukey comparisons were conducted when appropriate.

Statistical analyses of data were performed using SPSS (Version 28) and graphically presented using GraphPad Prism (Version 9.4). As indicated in Table S1, exploration of an elevated circle maze, three-chamber social approach, block chewing, novel object recognition, fear conditioning training and conditioned stimulus (CS) freezing were analyzed using a repeated measures (RM) ANOVA with genotype and sex as main effects. All other behavioral tests and select parameters that did not require statistical modeling using an RM ANOVA were analyzed using a multivariate ANOVA with genotype and sex as main effects. In cases in which significant genotype-by-sex interactions were detected, data were separately analyzed by sex; in the absence of significant genotype-by-sex interactions, genotype comparisons across groups were evaluated.

### Elevated circle maze

Animals at the age of 24 days were placed on an elevated circular platform with two walled regions (39 cm, height; 60 cm, diameter; 6.3 cm, platform width; 15 cm, closed zone wall height; 46 cm, closed zone wall length). Subjects were placed in the open region, allowed to roam freely and video recorded for 10 min. Using open access event logging software [Behavioral Observation Research Interactive Software (BORIS); Friard and Gamba, 2016], a trained observer blinded to genotypes scored the recordings for time spent in the closed and open regions. The number of transitions between and the time spent within both regions was exported from the BORIS system. An entrance into either region was defined as a rat having at least half of its anterior torso in the given area.

### Three-chamber social approach

Animals at the age of 25 days were tested in a three-chamber apparatus with perforated walls to allow for interaction between subject and partner rats. A center chamber contained removal doors to two flanking side chambers of equal dimensions (42.5 cm, length; 17.5 cm, width; 23 cm, height). The two side chambers contained a perforated plexiglass wall to allow for indirect contact with the novel partner or object behind it. Two sessions were conducted for each subject animal; first, a habitation phase of 10 min allowing acclimation to the apparatus, and a second 10 min phase during which a novel conspecific sex-matched partner was placed randomly into one of the side chambers with a novel object placed in the other. The subject was allowed 10 min to freely explore the apparatus, and indirect social approach was scored using BORIS by a blinded scorer for time spent in each chamber and time spent directly at each perforated partition.

### Direct social interaction

At 26 days, subjects were single housed overnight in clean standard rat housing cages (40.6 cm, length; 20.3 cm, width; 19 cm, height) with only Sanichip bedding, free of enrichment. The following day, the subject's cage was placed inside a sound-attenuating chamber along with a wild-type same-sex conspecific partner. For 10 min, the interaction allowing direct contact was video recorded. A highly trained blinded observer scored the 10 min interaction for juvenile social play behaviors using the BORIS system. Observations scored included the duration and number of events of play behavior (combination of nape/pounce, wrestling and pinning behaviors), sniff and following of the conspecific, and paw-to-torso contact towards the conspecific. The aggregate sums of duration and events across all categories were calculated and classified as active direct social behaviors.

### Open-field assay

At 27 days, subjects were allowed to explore an open-field apparatus (60 cm, length; 60 cm, width; 30 cm, height) for 15 min. Several parameters were measured via a Fusion v5.3 SuperFlex Open Field System (Omnitech Electronics, Columbus, OH, USA), which detects movement by breaks in infrared beams such as horizontal and vertical activity, center distance as well as measurements of time spent in the center of the open field. According to the Fusion v5.3 manual, a 20.32 cm×20.32 cm region in the middle of the open field is defined as the ‘center’, with the area surrounding it as the ‘margin’.

### Acoustic startle response (ASR) and PPI of the ASR

At 28 days, subjects were evaluated for sensorimotor gating using an SR-Lab System (San Diego Instruments, San Diego, CA, USA). Each subject was tested individually; at testing, animals were placed in plexiglass restrainers containing a force meter and positioned within the center of a soundproof chamber. An acclimation phase of 5 min with 70 dB white noise preceded each test session. Each test session consisted of 48 trials of randomized presentations of maximum-decibel sound of 120 dB to measure the baseline ASR, and presentations of three prepulses of 74 dB, 78 dB and 82 dB preceding the maximum-decibel sound. Percentage PPI of the ASR was calculated as 100−[(prepulse+maximum startle stimulus response/maximum startle response alone)×100], with the average of all three PPI levels used to calculate the global average PPI percentage.

### Marble burying

At 29 days, subjects were placed in a rat cage filled with corn cob bedding (∼10 cm) containing 20 marbles arranged in a 4×5 matrix and allowed to explore for 30 min. After the testing session, animals were removed, and the number of marbles visible, half-buried and three-quarters buried were counted. The number of marbles at least half-buried was used as a measurement of novelty-induced repetitive-like burying behavior.

### Novel object recognition

At 30 days, subjects were habituated to an empty plexiglass open field (60 cm, length; 60 cm, width; 30 cm, height) for 5 min and subsequently placed in an identical open field containing two identical unfamiliar objects (Lego; 6.5 cm, length; 6.5 cm, width; 16.5 cm, height) placed catercorner from each other for another 5 min. Subjects were video recorded during the exposure trial to the two objects. The next day, the same subjects were again habituated in an empty open field for 5 min and placed into the test arena, with one of the previous objects replaced randomly with a novel object. The trial session was video recorded, and a blinded scorer recorded the time the subject spent at each of the two objects. The novel object recognition index was determined by the time spent at the novel object versus the total time between the two objects [(time at novel object/total time spent at both novel and familiar objects)×100].

### Fear conditioning

On days 32 and 33, subjects were tested in a classical fear conditioning paradigm. The animals were placed in a metal bar-bottomed chamber (26 cm, length; 30 cm, width; 20 cm, height) for 120 s followed immediately by a 30 s period with 85 dB noise, ending with a 2 s, 1 mA foot shock. The following day, the subjects were placed back into the original chamber for 300 s to assess contextual fear memory. To evaluate cued fear memory, the chamber was cleaned, and black plexiglass was inserted into the chamber along with an artificial vanilla scent to generate a novel environment. Subjects were returned to the modified chambers for 180 s, followed by a conditioned stimulus of 85 dB for the remaining 180 s. Freezing behavior, a surrogate readout of memory, was measured for both contextual and cued fear memory. Freezing behavior was expressed as a percentage of time relative to the total test duration in which the subjects were motionless for >1 s; this was determined using automated detection software enabling video capture of frame-to-frame changes in movement (FreezeFrame Version 4.104, Actimetrics, Wilmette, IL, USA).

### Block-chewing test

At 34 days, subjects were single housed without environmental enrichment for 24 hours with access to a single wooden block (Aspen wood; 0.4 cm, length; 0.2 cm, width; 0.2 cm, height). Weight of the wooden block was measured before and after placement within each subject's cage. The difference in wooden block weight was taken to correspond as a measure of perseverative-like behavior.

### Hot-plate test

At 35 days, subjects were tested for pain sensitivity to thermal heat. Subjects were placed on a specialized test chamber containing a plate heated to 55°C with a plexiglass chamber surrounding the test area. The time to the first instance of shaking or licking of the hind paws was recorded, and provided a measure of latency to first response. Subjects were immediately removed from the test chamber upon observation of the first response and returned to their housing configuration.

### Overview of peripheral organ system assessments and statistical analyses of data

Rats heterozygous for the paternally inherited *Magel2* mutation (m+/p−) and rats homozygous with biparental inheritance of the *Magel2* mutation (m−/p−) were evaluated for alterations in body weight and composition, cardiac structure and function, and breathing patterns. For all physiological measures examined in paternally inherited *Magel2^m+/p−^* rats except breathing parameters, *n*=9-11 rats per genotype per group were tested; for breathing studies, *n*=6-14 rats per genotype per group were tested. For all physiological measures examined in homozygous *Magel2^m−/p−^* rats except breathing parameters, *n*=11-18 rats per genotype per group were tested; for breathing studies, *n*=10-12 rats per genotype per group were tested. All data were analyzed and represented as individual units within the violin plot-formatted graphs. Statistical analyses of data were performed using SPSS (Version 28) and graphically presented using GraphPad Prism (Version 9.4). As indicated in Table S2, all data were analyzed using a two-way ANOVA with genotype and sex as main effects. Parameters showing genotype-by-sex interactions were analyzed with genotype differences examined within sex. In the absence of significant genotype-by-sex interactions, data were analyzed with genotype as the main effect. With the exception of body weight, body composition measures were collected from m+/p− rats at 1 and 3 months of age, and m−/p− rats at 1 month of age.

### Dual-energy x-ray absorptiometry (DEXA)

To ascertain the body composition of rats mutant for *Magel2*, weights were first manually recorded, and individual rats were anesthetized with 2.5-3.5% isoflurane with 0.5 l/min of 100% O_2_ and placed ventral side down inside of a Faxitron^®^ UltraFocus^DXA^ system with a nose cone to maintain isoflurane flow. The limbs and tail were spread away from the torso to capture clean scans with the spine straightened. Parameters including bone mineral content, bone mineral density, fat weight/percentage, lean muscle weight/percentage and total weight were collected as an output text file for each rat.

### Quantitative magnetic resonance imaging (MRI)

Owing to the size of the rats at 3 months of age, DEXA using the Faxitron^®^ could not be performed. Instead, body compositions were acquired through an EchoMRI^TM^ Body Composition Analyzer using quantitative magnetic resonance technology. Weights of each rat were manually recorded before being placed into the EchoMRI^TM^ machine. Each subject rat was placed into and gently guided to end of the 700 g holder, with a Velcro barrier placed to minimize movement. A noninvasive scan was then performed on each rat while awake to obtain measurements of fat and lean muscle mass.

### High-frequency ultrasound echocardiogram

On the day prior to testing, each rat was fully anesthetized with isoflurane and placed on a heating pad to maintain body temperature, and the ventral region from its neck to xiphoid process was removed of hair using Nair^TM^ and a piece of gauze. The subsequent day, rats were anesthetized with 2.5% isoflurane mixed with 0.5 l/min of 100% O_2_ and placed ventral side up on an electric heating pad. Body temperature was continuously monitored throughout the procedure with a lubricated rectal probe and maintained between 36.5°C and 37.5°C. Pre-warmed echocardiography gel was applied to the hairless region above the heart. High-frequency ultrasound echocardiography was performed using a VisualSonics Vevo^®^ 2100 Imaging System with a MS250 transducer attached (FUJIFILM VisualSonics, Toronto, ON, Canada) to acquire ultrasound-based images of the left ventricle. Cross-sectional views of each rat's heart were generated using the parasternal short-axis mode, and data were acquired by recording from the papillary muscles.

### Surface electrocardiography

To measure the electrical properties of the rhythmic firing pattern of the left ventricle, we utilized an Indus Rodent Surgical Monitor^+^. Rats were anesthetized using 2.5% isoflurane with 0.5 l/min of 100% O_2_ and positioned ventral side down onto the heated surgical platform. Electrode cream was placed on each of the fore and hind paws, which were taped in place over individual leads. Following each cardiac cycle, amplitude and time of the P, Q, R, S and T waveforms, QT interval, and ST segment height were recorded and averaged; ventricular repolarization parameters like the JT interval were also calculated. Each rat tested had at least 1000 cardiac cycles measured, of which the above parameters were averaged across. Arrhythmic activity was monitored during each session by the trained experimenter.

### Whole-body unrestrained plethysmography

Rats were taken to the test room, their body weights were recorded, and they were then habituated to the testing room for 30 min. After the habituation period, each animal was placed within the plethysmography recording chambers, and breathing was recorded with synchronized capture of video for 1 h using data collection software (Ponemah 5.2 with Noldus Media Recorder, Data Sciences International). After testing, animals were returned to their home cages. Videos were scored by an automated algorithm to identify periods of calm state, and breathing signals were scored by an automated algorithm to identify breaths. Summaries of the breath parameters for each animal while in a calm state were generated and used for analysis (Ward et al., 2020).

### hiPSC culture and protein lysate collection

All hiPSCs were maintained on GelTrex (Thermo Fisher Scientific) in mTeSR Plus (Stem Cell Technologies) or in StemMACS iPS-Brew XF (Milteny Biotec) with medium change every other day and kept in a 37°C incubator with 5% CO_2_. hiPSCs were regularly split at ∼80% confluency or earlier based on morphology with Versene (Gibco) treatment. For total protein extracts, cells were harvested, washed with PBS and lysed in RIPA buffer containing Halt Protease-Inhibitor-Cocktail (Thermo Fisher Scientific) for 1 h on ice. Quantity of protein was assessed by a Pierce™ BCA Protein Assay Kit (Thermo Fisher Scientific), according to the manufacturer's instructions.

### Mass spectrometry for MAGEL2 wild-type and mutant truncated species

Mass spectrometry experiments were conducted through the Baylor College of Medicine Mass Spectrometry Proteomics Core. For each animal, a 5 mm^3^ section of rat hypothalamus was dissected, flash frozen in liquid nitrogen and crushed to powder with a pestle on a liquid nitrogen chilled steel block. Powdered tissues were lysed on ice for 30 min with 400 ml RIPA buffer (50 mM Tris-HCl pH 7.6, 150 mM NaCl, 1 mM EDTA, 0.25% sodium doxycholate, 1% NP-40, 0.1% SDS), supplemented with protease (GeneDepot, P3100) and phosphatase (homemade; 10 mM NaF, 1 mM Na_3_VO_4_, 10 mM β-glycerolphosphate) inhibitors. The lysates were further sonicated (GE505 Ultrasonic Processor) three times with 10 s ON and 30 s OFF intervals on 20% power on ice and cleared by centrifugation at 21,000 ***g*** (15,000 rpm) for 20 min at 4°C (Eppendorf 5424R Centrifuge). Then, 50 µg of supernatant was mixed with NuPAGE LDS Sample Buffer (Thermo Fisher Scientific, NP0007) and boiled at 90°C for 10 min. The proteins were separated on pre-cast NuPAGE Bis-Tris 10% protein gel (Invitrogen, NP0301BOX). Gels were stained with Coomassie (0.025% Brilliant Blue R-250, 40% MeOH, 7% AcOH), and two bands were excised corresponding to 93-170 kDa and 53-93 kDa. The expected molecular mass (MW) of wild-type rat MAGEL2 is ∼125 kDa, with predicted localization within the 93-170 kDa region, while the predicted ∼71.5 kDa MW of a truncated MAGEL2 would localize to the 53-93 kDa region of the gel. The bands were then crushed and digested with 22 ml trypsin solution (20 ml of 50 mM ammonium bicarbonate and 2 ml of 100 ng/ml trypsin; GenDepot, T9600) overnight at 37°C with shaking. For mass spectrometry runs, excised peptides were re-suspended in 5% methanol+0.1% formic acid solution and measured on an Exploris Orbitrap 480 mass spectrometer (Thermo Fisher Scientific).

Predicted expression of MAGEL2 protein is very low; therefore, the mass spectrometry method included a data-dependent acquisition (DDA) experiment (top 20 most intense ions with a dynamic exclusion duration of 15 s) and a targeted experiment for predicted peptides of MAGEL2. As empirical data on behavior of peptides from wild-type rat MAGEL2 were not available to evaluate potential best responders, 18 peptides were chosen based on their theoretical suitability for detection. A possible truncated MAGEL2 species was evaluated based on predicted peptides following trypsin digestion, similar to the wild-type approach. Raw data were searched against a rat RefSeq RefProtDB. The Proteome Discoverer software suite (PD Version 2.0.0.802; Thermo Fisher Scientific) was used to search the raw files with the Mascot search engine (v2.5.1, Matrix Science), validated peptides with Percolator v2.05 and provide MS1 quantification through the Area Detector Module. All peptide spectral matches for MAGEL2 were manually evaluated.

For hiPSCs, 20 µg cell lysate was resolved on pre-cast NuPAGE Bis-Tris 10% protein gel. Two band regions were cut as follows: band 1 for 60-90 kDa region and band 2 for 120-170 kDa region. In-gel digestion and peptide extraction was carried out as described before. The peptides were analyzed on a nano-LC 1000 system (Thermo Fisher Scientific) coupled to an Orbitrap Fusion mass spectrometer (Thermo Fisher Scientific). The nanoLC column and gradient settings were the same as described previously for the rat mass spectrometry experiments. The mass spectrometry instrument was operated in DDA mode with a targeted experiment for predicted MAGEL2 peptides in wild types and mutants. Peptides were selected based on their uniqueness in each of the genotype. The targeted experiment was performed in IonTrap [32% higher-energy C-trap dissociation (HCD)] with a 1.6 m/z isolation window in the quadrupole. The mass spectrometry raw data were searched against human NCBI RefSeq protein database (updated 24 March 2020) in Proteome Discoverer software suite. Precursor mass tolerance was set to 20 ppm and fragment ion tolerance to 0.5 Da. All peptide spectral matches for MAGEL2 were manually evaluated. The MS1 and MS2 peaks were extracted using Skyline software (MacCoss laboratory, University of Washington).

## Supplementary Material

10.1242/dmm.049829_sup1 Supplementary information
