# Supplementary material for: Magel2 truncation alters select behavioral and physiological outcomes in a rat model of Schaaf-Yang syndrome
Source: Dis Model Mech. 2023 Feb 3;16(2):dmm049829. doi: 10.1242/dmm.049829 (PMC9922728; doi:10.1242/dmm.049829)
Supplement: Supplementary information [file dmm-16-049829-s1.pdf]

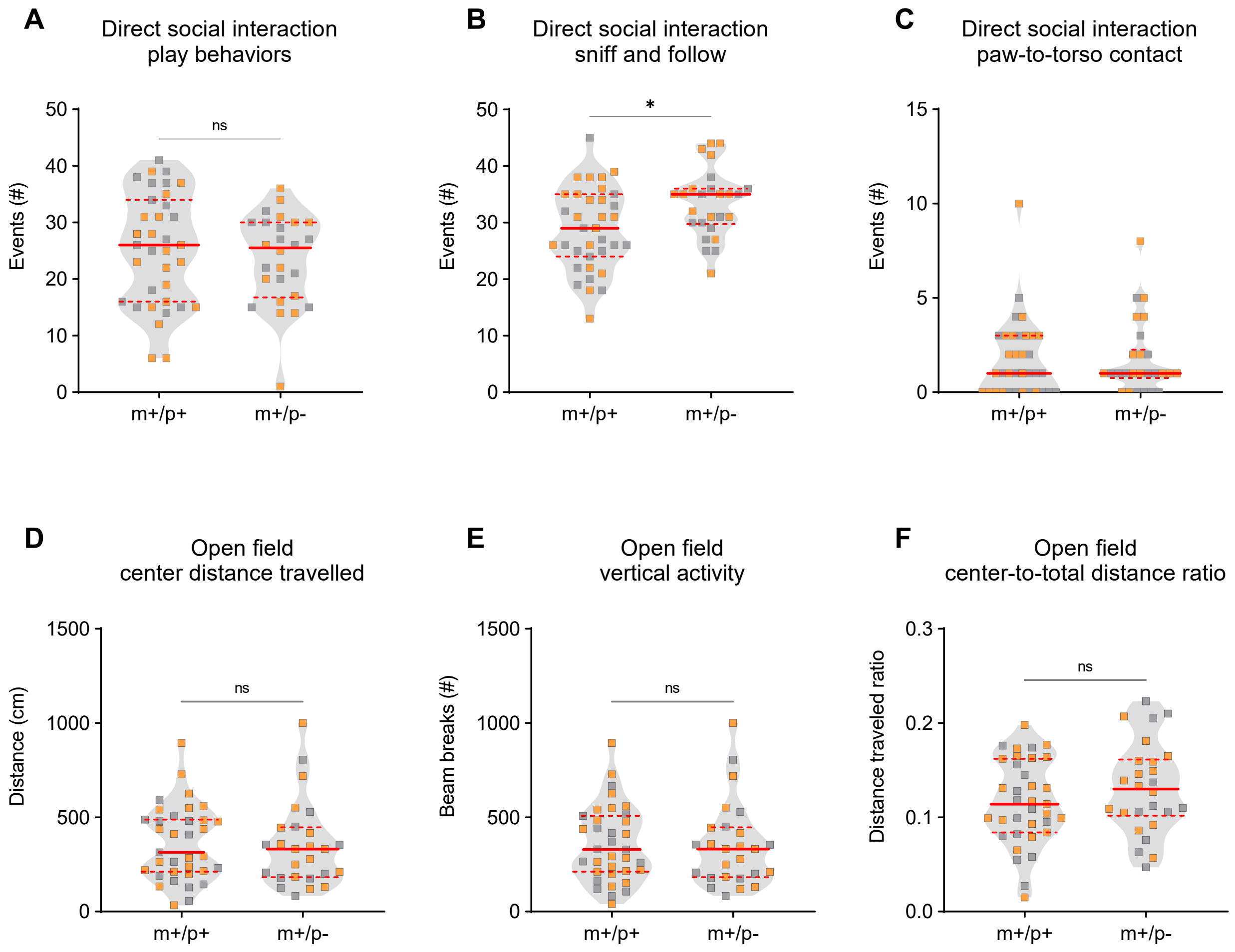

Fig. S1. *Magel2*$^{m+/p-}$ rats display select alterations in dyadic social interaction, and normal performance in select parameters of the open field test.

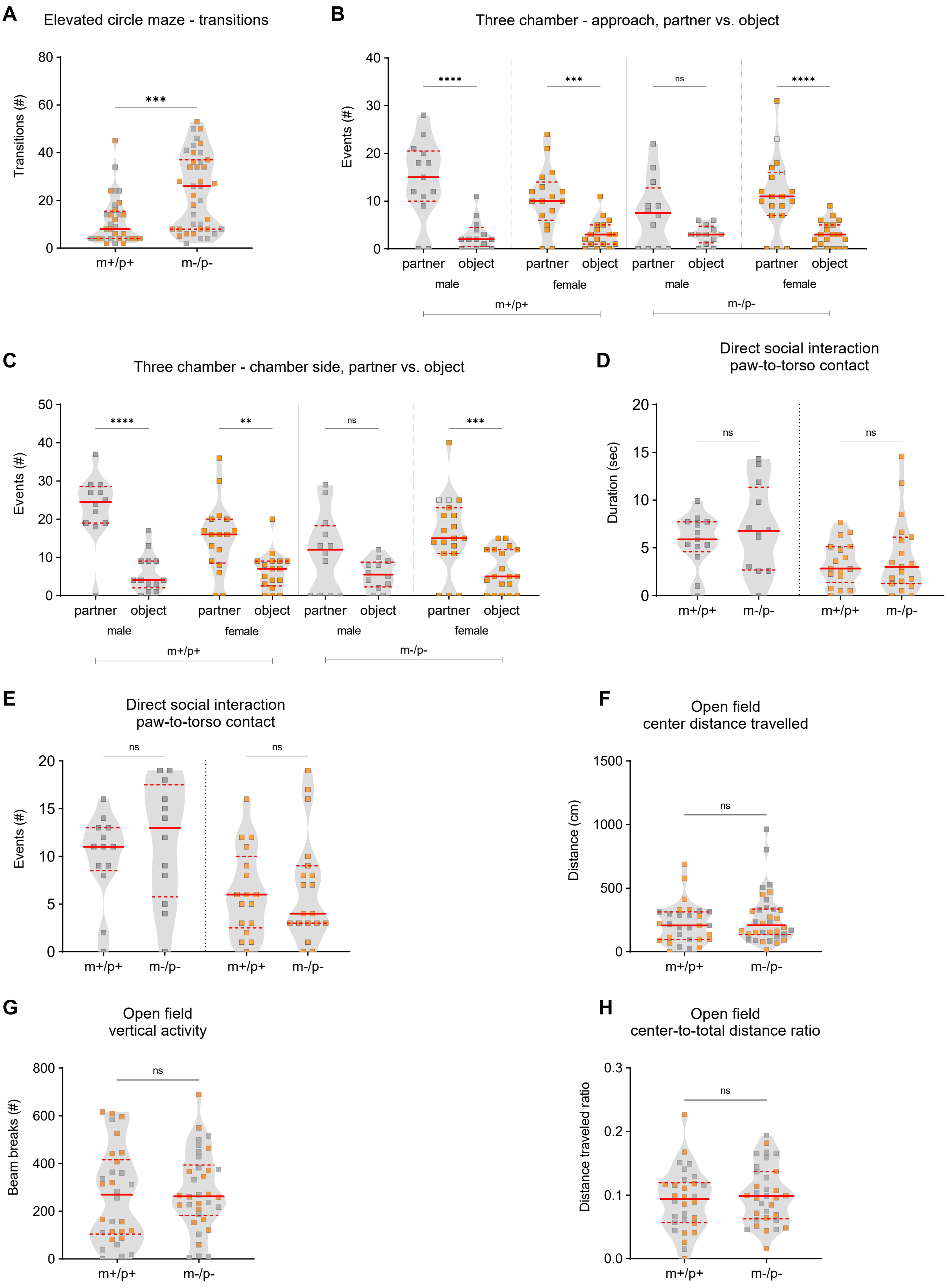

Fig. S2. *Magel2*$^{m-/p-}$ rats display additional select alterations in anxiety-like behavior and social approach, normal generalized contact in dyadic social interaction tests, and normal performance in select parameters of the open field test.

Disease Models & Mechanisms • Supplementary information

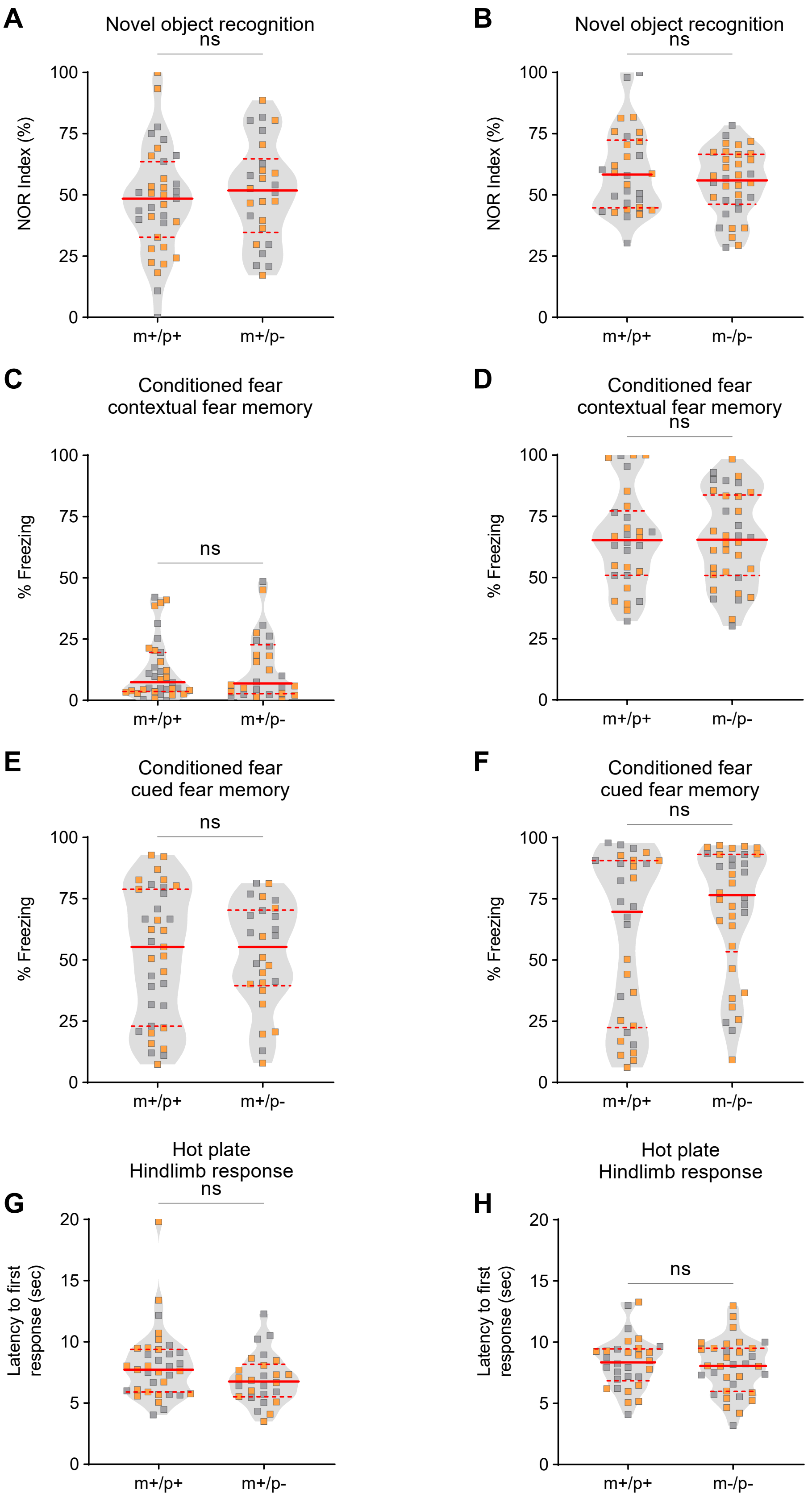

Fig. S3. Novel object recognition, aversive fear memory and pain sensitivity do not appear altered in *Magel2* mutant rats.

Disease Models & Mechanisms • Supplementary information

Table S1. Statistical summary of behavioral data

Click here to download Table S1

Table S2. Statistical summary of organ systems physiology data

Click here to download Table S2

Disease Models & Mechanisms • Supplementary information
